# The Anti-Hiv Candidate Abx464 Dampens Intestinal Inflammation by Triggering Il-22 Production in Activated Macrophages

**DOI:** 10.1038/s41598-017-04071-3

**Published:** 2017-07-07

**Authors:** Karim Chebli, Laura Papon, Conception Paul, Aude Garcel, Noëlie Campos, Didier Scherrer, Hartmut J. Ehrlich, Michael Hahne, Jamal Tazi

**Affiliations:** 1 IGMM, CNRS, Univ. Montpellier, Montpellier, France, 1919 route de Mende, 34293 Montpellier Cedex 5 Montpellier, France; 2 ABIVAX, 1919 route de Mende, 34293 Montpellier Cedex 5 Montpellier, France

## Abstract

The progression of human immunodeficiency virus (HIV) is associated with mucosal damage in the gastrointestinal (GI) tract. This damage enables bacterial translocation from the gut and leads to subsequent inflammation. Dextran sulfate sodium (DSS-exposure) is an established animal model for experimental colitis that was recently shown to recapitulate the link between GI-tract damage and pathogenic features of SIV infection. The current study tested the protective properties of ABX464, a first-in-class anti-HIV drug candidate currently in phase II clinical trials. ABX464 treatment strongly attenuated DSS-induced colitis in mice and produced a long-term protection against prolonged DSS-exposure after drug cessation. Consistently, ABX464 reduced the colonic production of the inflammatory cytokines IL-6 and TNFα as well as that of the chemoattractant MCP-1. However, RNA profiling analysis revealed the capacity of ABX464 to induce the expression of IL-22, a cytokine involved in colitis tissue repair, both in DSS-treated mice and in LPS-stimulated bone marrow-derived macrophages. Importantly, anti-IL-22 antibodies significantly reduced the protective effect of ABX464 on colitis in DSS-treated mice. Because reduced IL-22 production in the gut mucosa is an established factor of HIV and DSS-induced immunopathogenesis, our data suggest that the anti-inflammatory properties of ABX464 warrant exploration in both HIV and inflammatory ulcerative colitis (UC) disease.

## Introduction

ABX464 is a first-in-class antiviral drug candidate for treating patients infected with human immunodeficiency virus^1, 2^. ABX464 is an orally available, well-tolerated small molecule that blocks HIV replication through the modulation of RNA biogenesis^3^. ABX464 demonstrates anti-viral activity in treatment-naïve patients and induces a long-lasting control of the viral load in HIV-infected humanized mice after treatment arrest^3^. This treatment might improve current therapies by hampering the appearance of virus resistance and, more importantly, by leading to a long-lasting control of HIV.

Despite the successful control of viremia, many HIV-infected individuals treated with antiretroviral therapy (ART) exhibit residual inflammation^4^ associated with non-AIDS-related morbidity and mortality^5^. Several reports have shown that measures of inflammation and immune activation are the best independent predictors of disease progression in HIV-infected individuals^6^. It is also known that elevated inflammation and immune activation predict non-AIDS-associated morbidities and mortality, even in well-suppressed treated HIV-infected individuals^7^. Mucosal damage to the gastrointestinal (GI) tract with resulting microbial translocation significantly contributes to the heightened and persistent chronic inflammation and immune activation characteristic to HIV infection^6, 8, 9^ and might contribute to virus persistence during ART^10^. Recently, a dextran sulfate sodium (DSS)-induced nonhuman primate (NHP) colitis model in SIV-infected rhesus macaques a natural host species for SIV that does not manifest GI tract damage or chronic immune activation during infection, was used to demonstrate that DSS-exposure results in colitis with elevated levels of plasma SIV RNA, sCD14, LPS, CRP and mucosal CD4 T cell loss^9, 11^. The pathogenic potential of bacterial translocation has also been studied in humanized mice exposed to DSS to provide evidence that HIV chronic infection contributes to systemic inflammation under DSS-exposure^12, 13^. Given the similarities in colitis induction between DSS-exposed macaques and mice^11^, the antiviral activity of ABX464 in humanized mice model^3^ and its pharmacodynamics and pharmacoketics profiles in rodent, NHP^3^ and humans^2^, we tested the anti-inflammatory properties of this drug in DSS-treated mice. The results presented in this manuscript show for the first time that the antiviral drug ABX464 specifically acts on the immune system to attenuate mucosal disease induced by DSS.

## Results

**Figure 1:**
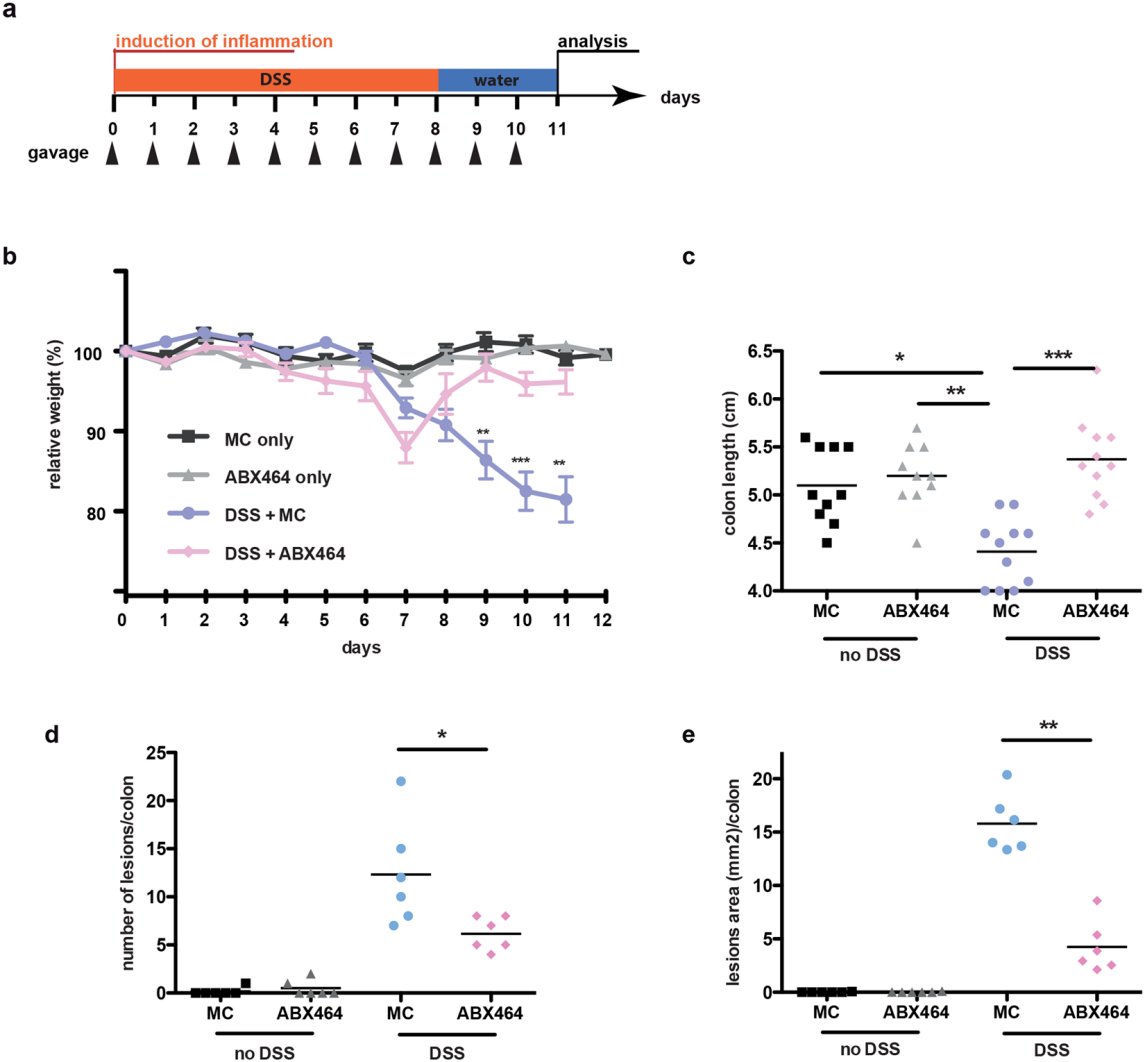
ABX464 treatment suppresses disease severity in DSS-induced colitis. (**a)** C57BL/6 mice (n = 12 each cohort) subjected to the DSS colitis protocol shown received orally once a day ABX464 (50 mg/kg) in methylcellulose or methylcellulose only through gavage. (**b)** Weight development in ABX464 and methycellulose (MC) only treated mice during DSS-induced colitis. Control cohorts included mice not exposed to DSS. (**c**–**e**) At the end of the protocol described in (**a**) mice were sacrificed and colons were analyzed by histology for colitis severity including number of lesion (**c**), lesion size (**d**) and colon size (**e**).

To test the anti-inflammatory properties of ABX464, we employed the commonly used DSS-induced experimental model of colitis. In this model inflammation is specifically induced in the colon via the administration of DSS in drinking water for approximately 5–8 days^14^. To test the effect of ABX464 in DSS-induced colitis, the compound was suspended in methylcellulose (MC), an established carrier for drug delivery to the colon^15^, and administered for 8 days via gavage (Fig. 1a). Mice receiving MC only served as a control. DSS-induced weight loss, an established symptom of intestinal injury, was significantly reduced in mice receiving ABX464 (Fig. 1b). This induced intestinal inflammation is usually strongest 3 days after the termination of the DSS challenge^14^. Strikingly, the weight of ABX464-treated mice had already returned to pre-treatment levels at that time point, and the mice displayed decreased disease parameters such as smaller and fewer colonic lesions as well as decreased shrinkage of colon length (Fig. 1c–e). Importantly, ABX464 did not affect the colons of mice not exposed to DSS (Fig. 1c–e). Noteworthy, the disease dampening influence of ABX464 in DSS-colitis was observed in experiments performed in different animal facilities, suggesting that this phenomenon is not dependent on a particular intestinal flora (see Methods section).

**Figure 2:**
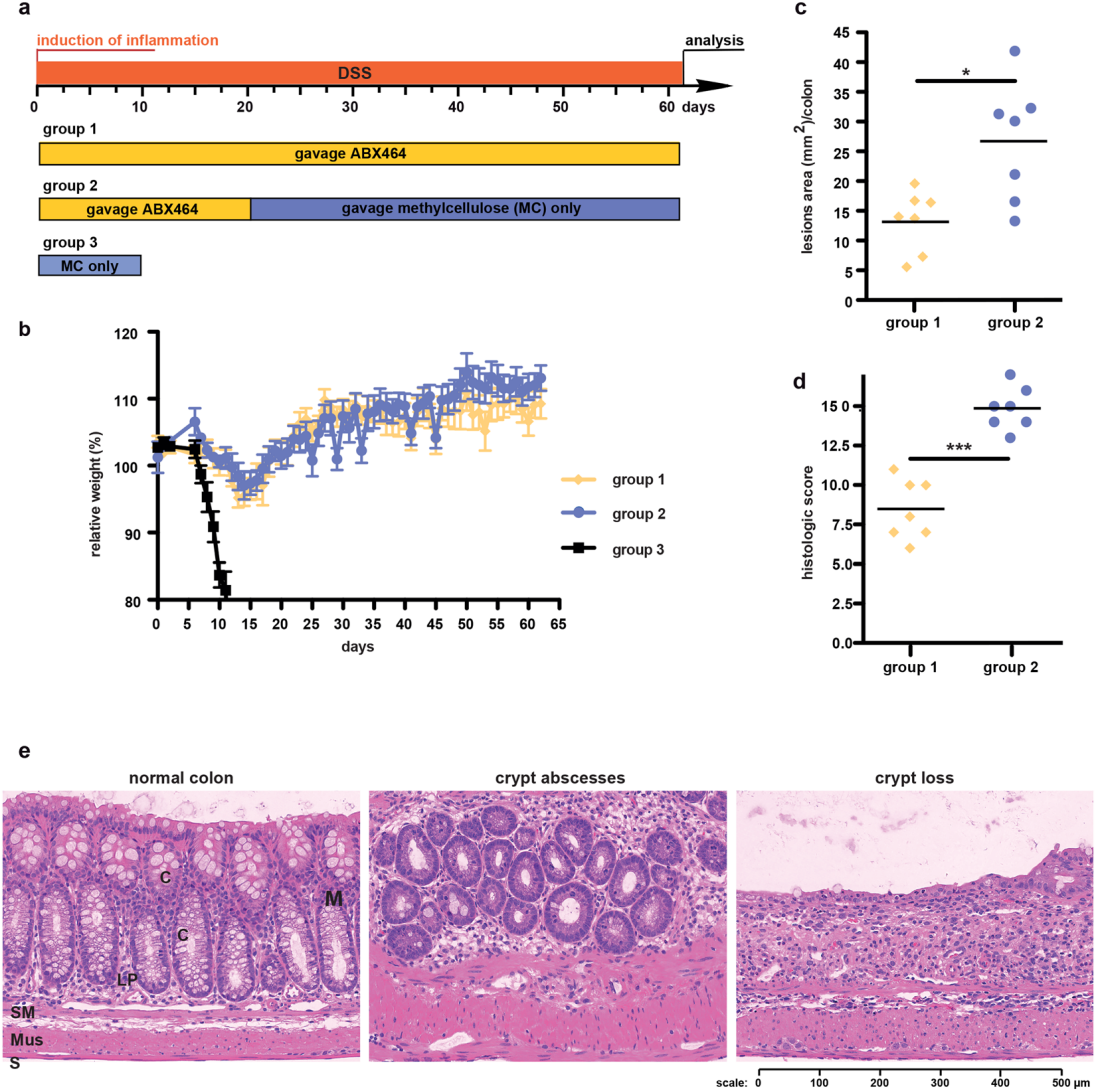
The protective effect of ABX464 is maintained in mice during continuous DSS-exposure. (**a**) C57BL/6 mice (n = 8 each cohort) were challenged with DSS for 63 days. Mice received orally once a day ABX464 in methylcellulose during the whole protocol (group 1) or only for the first 20 days followed by methylcellulose only administration (group 2). Mice of group 3 received methylcellulose during DSS-exposure and had to be scarified at day 10. (**b**) Weight development in ABX464-treated mice during DSS-administration. (**c,d**) Colons were analyzed by histology for lesion size (**c**) and colitis severity (**d**) at the end of the protocol. Histological scoring was performed as described in the Material and Methods section and detailed in Supplementary Table 1. (**e**) Representative images for normal colon, crypt abscesses and crypt loss, the latter two being part of the histological scoring shown in **2d**. Normal histology of the colon (normal colon); Mucosa, M, composed of epithelial crypt cells, C, underlined by lamina propria, LP; Submucosae, SM; Musculae, Mus; Serosa, S.

Next, we asked whether the protective effect of ABX464 is maintained during prolonged DSS exposure (Fig. 2a), which typically is lethal for mice. However, the daily application of ABX464 allowed the mice to be exposed to DSS for at least 63 days. Following a moderate initial body weight loss of approximately 5%, the mice recovered and maintained their initial body weight (Fig. 2b). Surprisingly, the mice that received ABX464 for only the first 20 days during the prolonged DSS exposure displayed similar body weight development and survival rates to those that received ABX464 for the entire study (Fig. 2b). Nevertheless, the colons of these two mouse cohorts displayed significant differences when examined for histopathological parameters including lesion size (Fig. 2c–e; Supplementary Table 1). This finding suggests that the 20-day administration of ABX464 provided a partially protective effect that was maintained for the duration of the 63-day DSS challenge.

**Figure 3:**
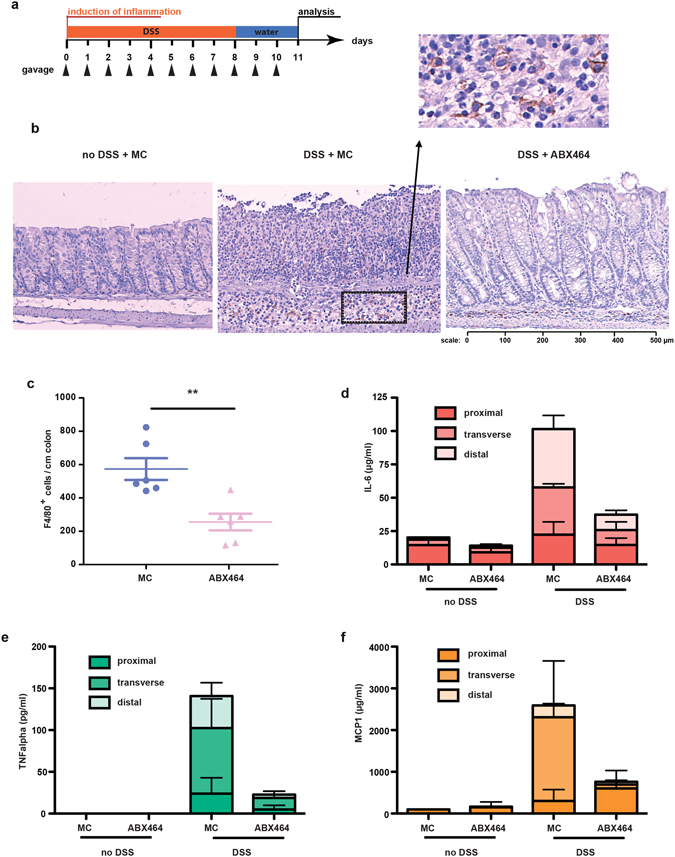
ABX464 decreases macrophage presence and pro-inflammatory cytokine production during acute colitis. **(a**) Acute colitis was induced as described in Fig. 1a. (**b**) Colons were analyzed for macrophage content by F4/80 labeling. Representative images of macrophage presence in colons of MC only treated mice (left hand panel), MC and ABX464 treated mice exposed to DSS are presented. (**c**) Total number of F4/80 + macrophages per colon per mouse are are shown (n = 8 each cohort). (**d–f**) Colons were taken at the end of the protocol, dissected in the proximal, middle and distal region and maintained in culture for 24 hours. Culture supernatant were analyzed for production of IL-6 (**d**), TNFalpha (**e**), and MCP-1 (**f**) by CBA as described in Methods section.

DSS exposure results in the chemical destruction of the colonic mucosal barrier with a subsequent increase in luminal bacterial translocation, thereby exposing the innate immune cells of the lamina propria to bacteria^16^. This effect leads to inflammation characterized by the infiltration of cytokine-producing neutrophils and macrophages^16^. In fact, ABX464-treated mice displayed fewer F4/80-positive macrophages in the colon (see Fig. 3a–c). Moreover, the colons of mice treated with ABX464 produced significantly less of the proinflammatory cytokines TNFα, IL-6 and monocyte chemoattractant protein-1 (MCP-1, CCL2) *ex vivo* (Fig. 3d–f). MCP-1 is a crucial chemokine that regulates the migration of macrophages and produced by different cell types including epithelial cells, fibroblasts and macrophages^17^. Our observation that colons isolated from ABX464-treated mice produce significantly less MCP-1 during inflammation might explain the decreased number of F4/80-positive macrophages.

**Figure 4:**
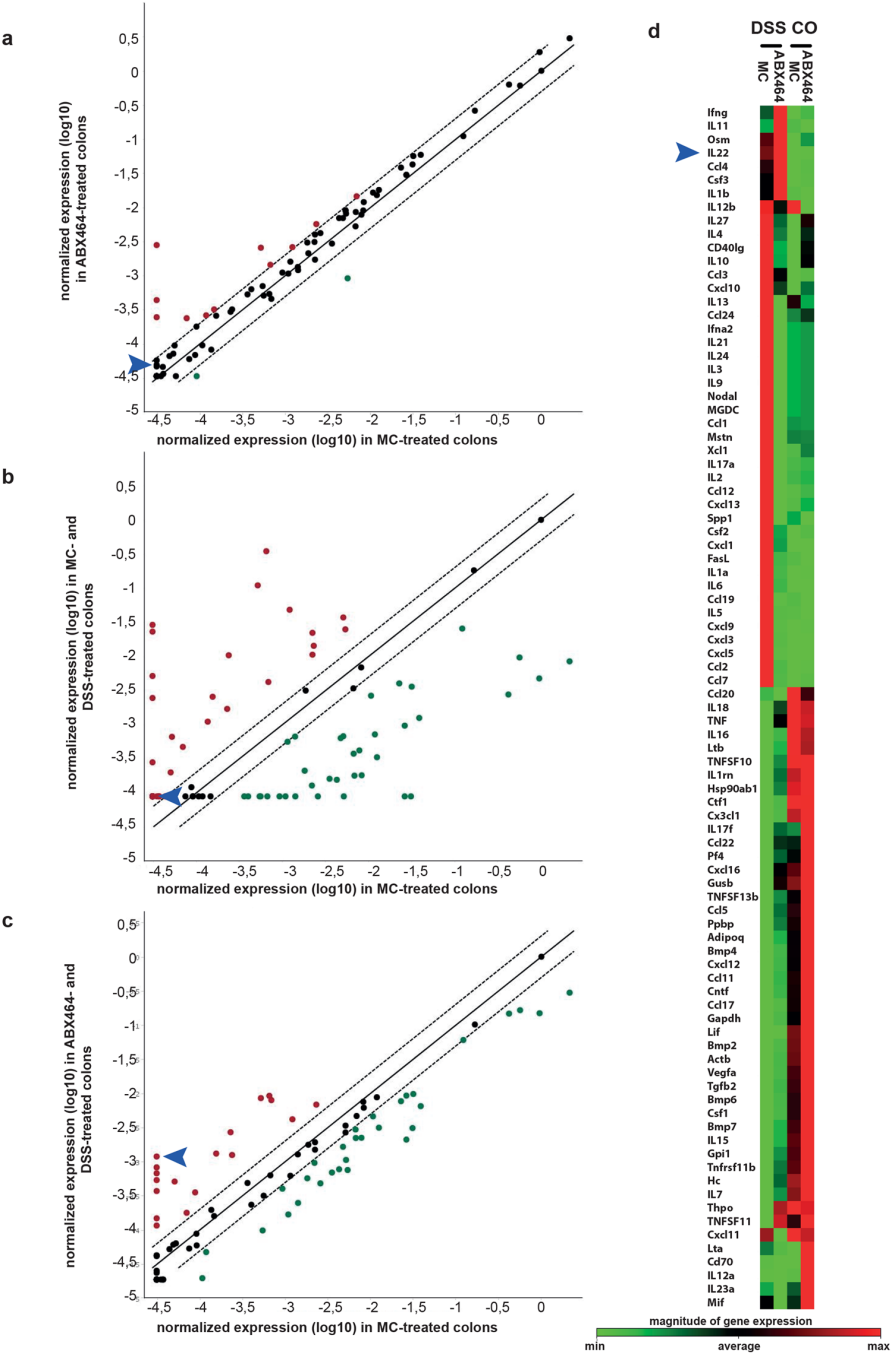
ABX464 treatment restores the expression of cytokine and chemokine signature in the colon of DSS-treated mice. (**a**) Identification of cytokines and chemokines regulated by ABX464 in colons of DSS-treated mice. RNA samples of from colons were taken at day 11 from mice exposed to DSS-exposure according to the protocol shown in Fig. 1a. Heatmaps show differential gene expression between colons of methylcellulose- and ABX464-treated mice as monitored by RT2 profiler PCR array (Qiagen). Scatterblots correlate gene expression in colons of ABX464-treated mice (**a**), methylcellulose (MC)- and DSS-exposed mice (**b**) and ABX464- and DSS-exposed mice (**c**) versus MC only treated mice. Samples represent the pool of RNA samples of n = 6 for each mouse cohort. (**d**) The clustergram shows differential gene expression between colons of MC- and ABX464-treated mice as monitored by RT2 profiler PCR array (Qiagen). Controls (Co) are tissue samples from mice not treated with DSS. Arrow heads indicate expression level of IL-22.

To elucidate the molecular responses modulated by ABX464, we performed quantitative polymerase chain reaction (qPCR) arrays to monitor the cytokine and chemokine expression in the colons of mice exposed or not to DSS in the presence or absence of ABX464. This molecule has no detectable effect on the expression profile of cytokines and chemokine signaling pathways in the absence of DSS exposure (Fig. 4a). Interestingly, ABX464 compensated for most of the expression differences induced by DSS exposure, which suggests that ABX464 restores the transcriptional program modified by DSS in the colon (Fig. 4 compare a, b and c). Only a few cytokines are up-regulated following ABX464 exposure in DSS-treated mice including IL-22^18^, a member of the IL-10 family (Fig. 4d).

**Figure 5:**
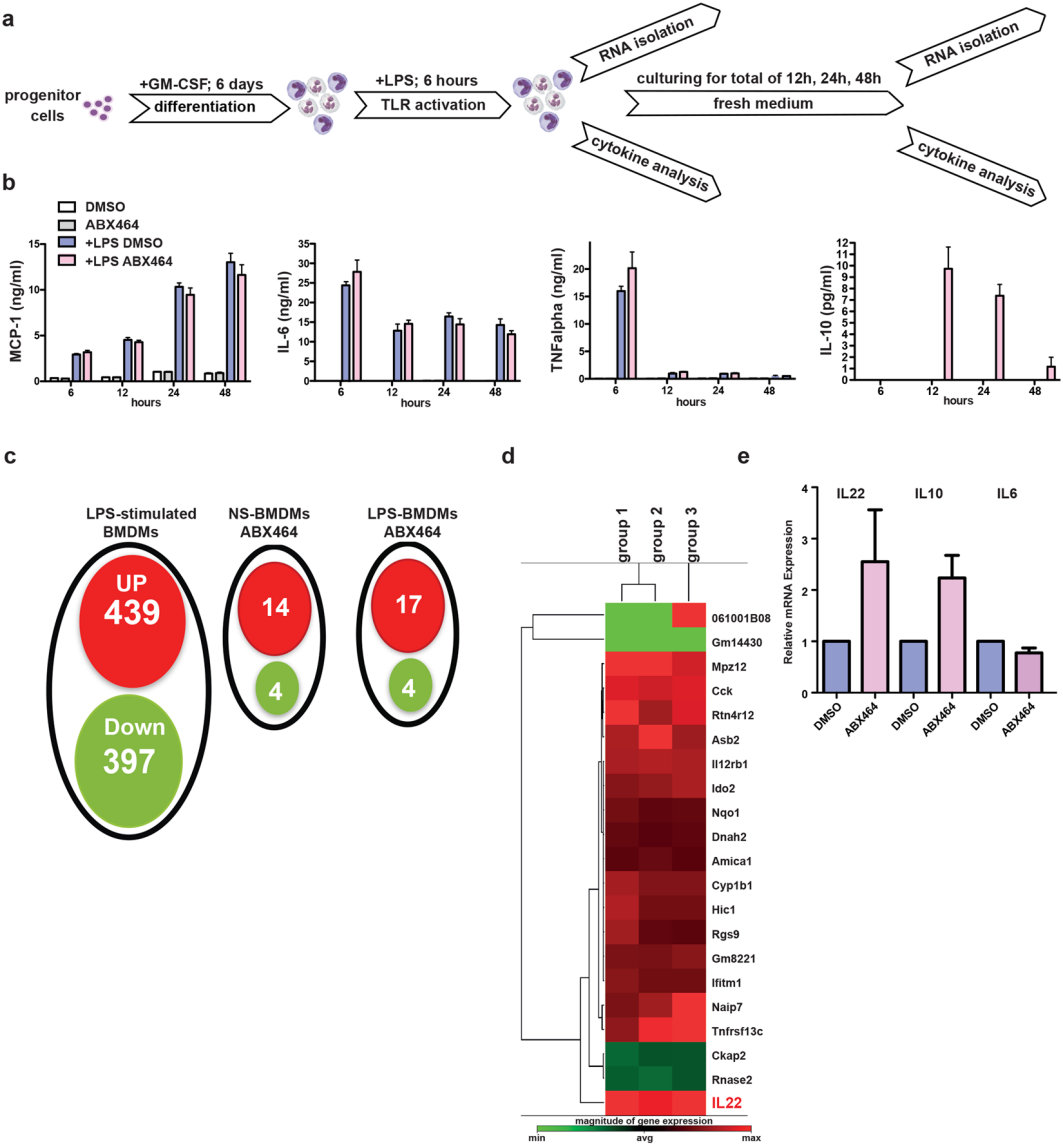
Effect of ABX464 on cytokine secretion and mRNA expression in BMDMs **(a**) Bone marrow isolated cells were cultured for 6 days in the presence of GM-CSF (50ng/ml) to differentiate into macrophages. Cells were kept in culture for additional 3 days in the presence of ABX464 (5 µM) or vehicle (DMSO) alone and for additional 6 hours stimulated with LPS (4 µg/ml). Cells were then kept in normal medium for additional 42 hours. Cell aliquots for RNA isolation and supernatants were taken at time 6, 12, 24 and 48 hours. **(b)** Culture supernatants of the indicated cell cultures were analyzed by CBA for the content of MCP-1, IL-6, TNFalpha and IL-10 as described in Materials and Methods. **(c)** A Venn diagram displaying the number of inducible or repressible (≥1.5 log2-fold) genes after 6 hours stimulation of LPS (LPS-stimulated BMDMs), non-stimulated BMDMs treated with ABX464 (NS-BMDMs ABX464) or LPS-stimulated BMDMs treated with ABX464 (LPS-BMDMs ABX464). **(d)** Identification of genes regulated by ABX464 in LPS-stimulated BMDMs. RNA samples of three group of cells taken at time point 12 h were analyzed by RNAseq and each group represents the relative expression between ABX464 and control (DMSO) treated samples. Only those genes are represented that were differentially expressed in each group. **(e)** Validation of elevated transcript levels of IL-22 in ABX464-treated LPS-stimulated BMDMs by qPCR.

The GI-tract homeostasis established by ABX464 in mice exposed to DSS suggests the presence of an active process that maintains the epithelial barrier. Intestinal macrophages are situated in the enteric luminal environment to appropriately respond to GI lesions and bacterial translocation^19^. Bone marrow derived macrophages (BMDMs) are a common cellular model used to study murine macrophages^20^. Therefore, we activated BMDMs with LPS for 6 h to mimic bacterial activation and treated the cell cultures with ABX464 to determine the direct effect of the drug candidate on the production of different inflammation-associated cytokines that macrophages can secrete (Fig. 5a). As expected, the stimulation of BMDMs with LPS for 6 h induced the expression of IL6, TNFα, and MCP1 (CCL2) but not that of IL10 (Fig. 5b). The expressions of MCP1 and IL6 persisted for 48 h post LPS-stimulation, whereas TNFα expression was down-regulated at 12 h (Fig. 5b). Strikingly, ABX464-exposed BMDMs displayed an increased production of IL-10 at 12 and 24 h post LPS-stimulation but did not alter levels of the pro-inflammatory cytokines IL6 and TNFα (Fig. 5b). The decreased production of IL-6, TNFα and MCP1 detected in ABX464-treated colons (Fig. 3d–f) is most likely associated with the decreased numbers of pro-inflammatory macrophages observed in those tissues, although other cellular components of the colon (e.g., epithelial and stromal cells) might also contribute (Fig. 3b). Moreover, ABX464 treatment had no detectable effect on the production of these cytokines in un-stimulated BMDMs, which suggests that ABX464 only acts on activated BMDMs (Fig. 5b). The induced expression of IL10 in LPS-stimulated BMDMs via ABX464 could contribute to its protective effect observed in DSS-treated mice. In fact, IL-10 plays a crucial role in intestinal homeostasis^21, 22^ because mice deficient in IL-10 or IL-10 receptor develop spontaneous colitis^21^, whereas mice with ectopic IL-10 expression in their intestinal epithelial cells are protected against DSS-induced acute colitis^23^.

We next performed a high-resolution transcriptome analysis of ABX464-treated BMDMs stimulated with LPS. Given that IL10 induction via ABX464 was only visible at 12 h and not 48 h post-stimulation with LPS, we analyzed the BMDMs stimulated with LPS for 6 h and then treated them with ABX464 without LPS for an additional 12 h. The data from three distinct group of BMDM cultures were combined and analyzed for different gene expression levels using RNAseq (the raw data are accessible from Gene Expression Omnibus repository, upon request). Consistent with previous reports^24^, the RNA-seq analysis revealed numerous differentially expressed genes (1383) in the LPS-stimulated BMDMs among which 439 and 397 genes increased and decreased in expression ≥ 1.5 log2-fold, respectively (Fig. 5c Supplementary Table S2). The inflammation- and immune response-related genes exhibited the most dramatic induction levels following the LPS challenge (Supplementary Table S2). However, the use of a high-stringency ABX464 treatment only modulated the expression of a limited number of genes in un-stimulated (18 genes; Fig. 5c and Supplementary Table S3) and LPS-stimulated BMDMs (21 genes, Supplementary Table S4), among which 6 genes were in common (Supplementary Table S4). The reduced number of differentially expressed genes revealed by this analysis shows that ABX464 had little effect on the global transcription changes induced by LPS. Interestingly, the only cytokine identified to be up-regulated by ABX464 in LPS-stimulated BMDMs was IL-22 (Fig. 5d, Supplemental Table S4), a key cytokine in intestinal inflammation recovery^25^. The qPCR analysis confirmed the induction of IL-22 via ABX464, and IL-10 was also up-regulated (Fig. 5e). In fact, the RNA-seq analysis detected increased IL-10 expression in only two of the three sample sets and was therefore not initially identified because of the stringency applied. The unaltered production of TNFα, IL-6 and MCP-1 and the up-regulation of IL-22 and IL-10 in LPS-stimulated BMDMs treated with ABX464 was finally confirmed using qPCR arrays to monitor cytokine and chemokine expression (Supplementary Fig. S1). Importantly, ABX464 did not induce IL-22 or IL-10 production in un-stimulated BMDMs (Supplementary Fig. S1).

**Figure 6:**
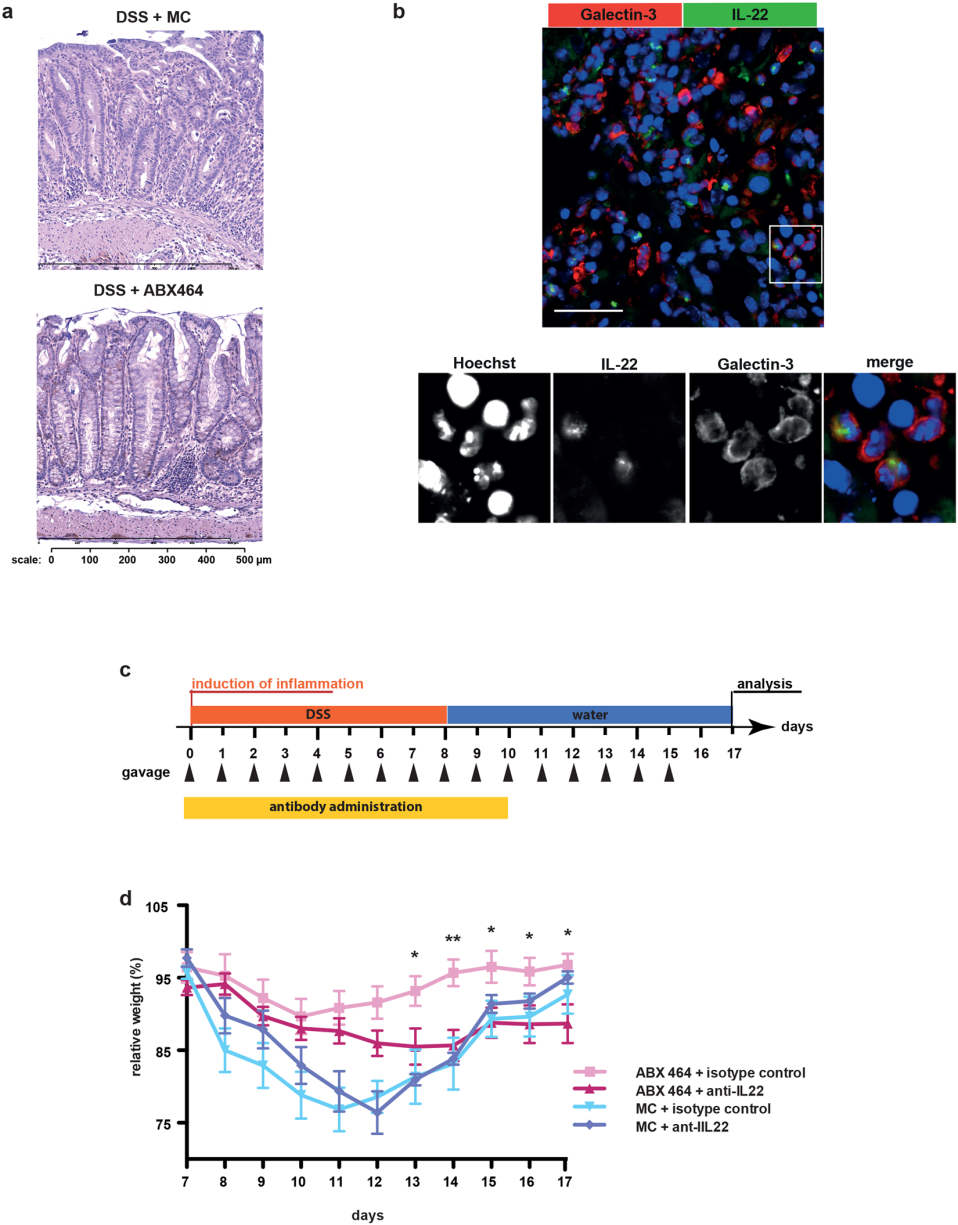
The colitis dampening effect of ABX464 is partially mediated by IL-22 **(a)** Elevated protein levels of IL-22 are detectable on paraffin sections of colons taken from ABX464 treated mice. Colons were isolated at day 11 from mice exposed to DSS-exposure according to the protocol shown in Fig. 1a. (**b**) Confocal images of paraffin-sections from a colon of an ABX464-treated mouse as described in Fig. 6a. Section was stained for galactin-3 to identify macrophages (red) and IL-22 (green). Nuclei were stained with Hoechst (blue). The selected images shown are representative. Scale bars represent 100 µm the panel show 4x blow-ups. The cells shown in the blow up are located in the lamina propria between perturbed crypts. (**c**) C57BL/6 mice (n = 7 each cohort) were subjected to the DSS colitis protocol shown. received orally once a day ABX464 in methylcellulose or methylcellulose (MC) only through gavage. ABX464 and MC-treated were divided into two groups receiving daily 100 µg of either antagonistic anti-IL-22 antibody or isotype control. **(d)** Weight development in ABX464 and methycellulose (MC) only treated mice during DSS-induced colitis in the presence of either control or anti-IL-22 antibody.

IL-22 is a cytokine that regulates tissue repair and recovery^26–28^; it was also recently shown to ameliorate intestinal inflammation in a mouse model of ulcerative colitis^25^. Besides, IL-22 can also induce the enhanced expression of IL10^29^. Notably, our immunohistological analysis of DSS-treated mice confirmed that the IL-22 expression in the colon was significantly up-regulated by ABX464 prior to the recovery phase (i.e., after the termination of DSS, Fig. 6a). Immunofluorescence (IF) analysis revealed that a subpopulation of IL-22 positive cells expresses galactin-3 (Mac-2) a marker of macrophages (Fig. 6b), which, however, was reported to be expressed by other inflammatory immune cells such as dendritic cells^30–32^. This result prompted us to determine whether IL-22 mediated the protective effect of ABX464 in this colitis model. To this end, an inhibitory anti-IL-22 antibody was administered during the recovery phase (Fig. 6c). We found that treatment with the anti-IL-22 antibody significantly reduced the protective effect of ABX464 from DSS-induced acute colitis as judged by body weight loss (Fig. 6d).

## Discussion

Taken together, this study revealed the capacity of the anti-HIV drug candidate ABX464 to dampen intestinal inflammation in mice exposed to DSS at least partially by triggering the secretion of IL-22; macrophages are an important source of this cytokine. Through its anti-inflammatory effect, ABX464 might be able to modulate inflammatory ulcerative colitis (UC) disease in patients. While DSS-induced colitis can be used as a relevant model for the translation of mice data to human disease^14, 33^ further studies are needed to determine whether the protective effects of ABX464 against colonic inflammation is confined to DSS or can be generalized to other models of intestinal injury using either administration of other chemicals like trinitrobenzene sulfonic acid (TNBS), oxazolone, acetic acid and sulfhydryl inhibitors or bacterial infection^14, 33, 34^. These models could provide additional valuable insights toward the treatment of UC patients with ABX464.

The administration of DSS in drinking water is expected to induce disruption of colonic epithelial barrier with subsequent exposure of innate immune cells to commensal bacteria which then triggers an innate immune response. Thus, intestinal microbiota may play an important modifying role in the susceptibility and responsiveness of DSS-induced injury in mice to ABX464. No significant differences were observed in the efficacy of ABX464 to ameliorate DSS-induced inflammation when experiments are conducted in two different experimental facilities, knowing that this could be a source of variation in the gut microbiome^33, 34^. A more rigorous analysis of gut microbiota under different setting is required to precisely determine whether alteration in microbiota will have an impact on ABX464 activity.

Exposure of mice to DSS is accompanied by a robust Th1-type immune response to eliminate infiltrating pathogens and promote tissue healing^35^. Mucosal macrophages may prime the local inflammatory response through both phagocytosis of DSS, activation by bacteria products^36, 37^ and secretion of pro inflammatory cytokines like TNFα, IL6 and the chemokine MCP1^38^. Selective depletion of macrophages by targeting the CSF1R leads to enhanced susceptibility to DSS-induced colitis^39, 40^, implying that macrophages play a key role in the repair of the injured colonic tissue. Our work highlights a novel mechanism by which a drug or drug candidate (ABX464) can engage macrophages into the repair process. Both *ex vivo* and *in vivo* ABX464 promote expression of IL-22 in macrophages under stimulation by LPS or by translocated pathogens in the gut. IL-22 is known to fulfill two functions 1) protection against bacterial pathogens by enhancing phagocytosis 2) stimulation of proliferation, differentiation, and migration of intestinal epithelial cells, which aids in wound repair and restitution of the barrier^25, 26^. This is to our knowledge the first report where IL-22 is shown to be produced by macrophages, while it is known to be produced by natural killer (NK) cells, Th17/Th22 CD4^+^ T cells, and innate lymphoid cells (ILCs)^18^. In agreement with other studies by Zindl *et al*.^41^ we confirmed that another important source of IL-22 are neutrophils Gr-1 + cells (data not shown). Macrophages and neutrophils are the major innate immune cells that are accumulating in the colon following the induction of intestinal injury by DSS to play an active role in resolution of inflammation^36, 42^, it remains to be determined how the production of IL-22 by those cells does improve tissue repair to mediate the beneficial effect of ABX464. Furthermore, it will be interesting to address, whether the decreased number of F4/80 + cells in ABX464 treated mice is due to an altered proliferation of resident macrophages or due to an increased inflow of (Ly6C+) myeloid cells as previously described^43–45^.

## Methods

### Animal experimentation

Mouse experiments were performed in strict accordance with the guidelines of the European Community (86/609/EEC) and the French National Committee (87/848) for the care and use of laboratory animals. The study plan was approved by the Institutional Review Board at the Animal Facility of the Institute de Génétique Moléculaire de Montpellier (IGMM) and the Regional Ethics Committee for Animal Experimentation of Languedoc-Roussillon (agreement n° CEEA-LR-12087).

The DSS experiments described in Fig. 1 were performed at Amylgen (*In vivo* drug discovery, Montferrier sur Lez, France), whereas those described in Figs 2, 3 and 6 were done at the animal facility of IGMM. For DSS-induced colitis, age- and sex-matched C57BL6 mice received one cycle of 2.5% (w/v) DSS administered via drinking water. Mice received 40 mg/kg ABX464 suspended in MC or MC only via gavage when indicated. For antagonizing IL-22 *in vivo*, mice received either blocking IL-22 antibody (Ebioscience; clone IL-22JOP) or isotype control (Ebioscience; clone eBR2a).

### Histology, immunohistochemistry and immunofluorescence microscopy

Mouse colons were fixed in formalin solution for 16 h. A histological examination was performed on paraffin-embedded sections counterstained with hematoxylin and eosin. Lesion size was determined using NDP viewer software. Immunohistochemistry was performed on paraffin-embedded tissues cut into 4-μm sections. After blocking with 10% goat serum, samples were incubated with primary antibody overnight at 4 °C using F4/80 (clone BM8, Hycult biotech) or galactin-3 (clone M3/38, ebioscience) for the detection of macrophages or anti-IL-22 (Abcam ab18564). Secondary antibodies were either HRP-coupled anti-rat or anti-rabbit IgGs, visualized with 3,3’-diaminobenzidine (DAB). Histologic grading of DSS-induced lesions was determined with blinded genotype by a pathologist. Scoring included crypt distorsion and loss, immune cell infiltration thickening of muscularis mucose and serosa as well as loss of goblet cells (Supplementary Table 1).

For immunofluorescence analysis primary antibodies were incubated with fluorescent-labeled secondary anti-mouse/rabbit antibodies (Vector Laboratories). DNA was stained with 20 µg/ml 4’,6’-diamidino-2-phenylindole (DAPI).

Fluorescent images were acquired on a brightfield microscope (Leica) using Metamorph software, or inverted Confocal SP5 (Leica) using the Leica LAS AF software. Images were processed with ImageJ. Fluorescent secondary antibodies were labeled with Alexa 488 and 647 fluorophores. Images were assembled and adjusted with Adobe Photoshop/ Illustrator.

### Statistical analyses

Mann-Whitney *U* tests followed by Bonferroni post hoc tests and area under the curve (AUC) analyses were performed using GraphPad Prism V.4.00 (GraphPad Software, San Diego, California, USA). Data are shown as the mean ± SEM. A value of p < 0.05 was considered significant; p < 0.01 was considered very significant; and p < 0.0001 was considered extremely significant.

### Cell and tissue culture and cytokine measurement

Cell isolates from the femurs of C57BL6 mice were cultured for 9 days in the presence of GM-CSF (50 ng/ml; Peprotech). Cell cultures were treated for the last 3 days with either ABX464 (5 µM) suspended in DMSO or DMSO alone. After additional stimulation for 6 h with LPS (4 µg/ml; Invivogen), the supernatants were analyzed for cytokine production using a cytometric bead array technology (CBA; Becton Dickinson). The detection limits were 5 pg/ml for IL-6, 52.7 pg/ml for MCP-1, 17.5 pg/ml for IL-10, 7.3 pg/ml for TNFα, and 10.7 pg/ml for IL12p70. Gene profiling of cell aliquots was performed using qPCR, RT2 profiler PCR array and RNAseq (BGI, Hong Kong; see below). In some experiments, the colons from DSS-treated mice were isolated, washed with PBS and taken in culture in DMEM (10% FCS) for 24 h before cytokine measurement via CBA.

### qPCR analysis

The transcript levels of ABX464-treated and untreated cells and tissues were first determined using pooled RNA samples with a RT2 profiler PCR array for cytokines and chemokines (Qiagen). Differentially expressed transcripts were then validated using qPCR in single samples. qPCR was performed using SYBR Green (Roche) with the primers listed in Supplemental Table S5. Briefly, 1 μg of RNA was used to generate 20 μl of complementary DNA. Next, the complementary DNA samples were diluted and then subjected to 45 cycles of PCR at 95 °C for 4 s, 65 °C for 10 s and 72 °C for 15 s. The internal controls used were β-glucuronidase (GUS), hypoxanthine guanine phosphoribosyl transferase 1 (HPRT 1) and TFIID TATA box binding protein (TBP). The reactions were performed using a LightCycler 480 (Roche) machine. The data are shown as relative units where control bands were given a value of 1, and experimental values represented the fold change over the control following normalization with the three housekeeping genes. All of the experiments were performed in duplicate at least three times. The list of primers used in qRT-PCR are indicated in Supplemental Table S6.

### RNAseq analysis

The methods and results of the RNAseq analysis are submitted to Scientific Data (Manchon *et al*. unpublished results). The raw data GSE97062 are accessible from Gene Expression Omnibus repository, upon request.

### Data availability

The datasets generated during and/or analysed during the current study are available from the corresponding author on reasonable request. All data generated or analysed during this study are included in this published article (and its Supplementary Information files). All materials, data and associated protocols are promptly available to others without preconditions.

## Electronic supplementary material

Supplemental information Chebli et al
