# Supplementary material for: The Anti-Hiv Candidate Abx464 Dampens Intestinal Inflammation by Triggering Il-22 Production in Activated Macrophages
Source: Sci Rep. 2017 Jul 7;7:4860. doi: 10.1038/s41598-017-04071-3 (PMC5501810; doi:10.1038/s41598-017-04071-3)
Supplement: Supplementary file 1 — Supplemental information Chebli et al [file 41598_2017_4071_MOESM1_ESM.pdf]

Supplemental information

THE ANTI-HIV CANDIDATE ABX464 DAMPENS INTESTINAL INFLAMMATION BY TRIGGERING IL22 PRODUCTION IN ACTIVATED MACROPHAGES

Karim Chebli[1 3], Laura Papon[1 3], Conception Paul[1], Aude Garcel [2], Noëlie Campos[2], Didier Scherrer[2], Hartmut Ehrlich[2], Michael Hahne[1*] and Jamal Tazi[1*]

1) IGMM, CNRS, Univ. Montpellier, Montpellier, France
   1919 route de Mende, 34293 Montpellier Cedex 5, France

2) ABIVAX, 1919 route de Mende, 34293 Montpellier Cedex 5, France

3) These authors contributed equally to this work

* These authors share senior and corresponding authorship:

Prof. Jamal Tazi, PhD; Dr. Michael Hahne, PhD

Institut de Génétique Moléculaire de Montpellier
1919 route de Mende
34293 Montpellier Cedex 5
France
E-mails: jamal.tazi@igmm.cnrs.fr
michael.hahne@igmm.cnrs.fr

Tel: (33) 4 34 35 96 85 / Fax: (33) 4 34 35 96 34

Group 2 Mouse N°	crypt distortion	immune cell infiltration	thickening of muscularis mucose	loss of goblet cells	crypt absesses	crypt loss	thickening of serosa	total score /mouse
1	2	3	2	0	0	3	3	13
2	2	3	3	1	1	3	3	16
3	2	3	3	1	1	3	2	15
4	2	3	3	0	0	3	3	14
5	2	3	2	1	1	2	4	15
6	2	3	3	1	2	3	3	17
7	2	3	3	0	1	3	2	14

Group 1 Mouse N°	crypt distortion	immune cell infiltration	thickening of muscularis mucose	loss of goblet cells	crypt absesses	crypt loss	thickening of serosa	total score /mouse
1	1	3	2	0	0	0	1	7
2	1	3	2	1	0	0	1	8
3	1	2	2	0	0	0	1	6
4	2	3	2	1	0	2	1	11
5	2	2	2	1	1	1	1	10
6	2	2	2	1	1	1	1	10
7	1	2	2	0	1	0	1	7

Supplementary table 1: Histological scoring of the colon taken from mice exposed to the protocol described in Figure 2A. The scoring for each parameter was performed by a pathologist from the histology platform RHEM in Montpellier. Values were from 0 to 3, with 0 being lowest and 3 highest level of alteration. The resulting values for total score are shown in Figure 2D.

Supplemental Table S2 : Up and down regulated genes in LPS-stimulated BMDMs. In Red are genes involved in inflammation and immune response

Symbol	Gene ID	Gene Length	log2 Ratio (LPS/NS)	Up-Regulation (LPS/NS)	P-value	FDR
Ksr2	75706	1689.00	9.28540221886225	Up	1.707192e-63	2.00444425411765e-62
Ccl5	20304	579.00	8.54896081454134	Up	0	0
Slc1a5	20511	2897.00	8.48049323416513	Up	0	0
Pyhin1	623121	2674.96	8.44914864537544	Up	4.1473e-220	1.27932894181818e-218
Itpripl1	320910	3096.00	8.26209484537018	Up	6.02554e-58	6.54058295841331e-57
Susd2	100041294	710.00	7.76155123244448	Up	1.77622e-09	5.98993212482608e-09
Npas4	18126	4164.00	7.68233626971138	Up	0	0
Ms4a6b	64380	1723.00	7.4412070522599	Up	0	0
Apol9b	71898	1516.89	7.22363046744425	Up	5.52282e-169	1.37794359e-167
Tgtp2	21822	2809.00	7.18982455888002	Up	3.03606e-25	1.80672725219221e-24
1110032F04Rik	68725	2578.00	7.04439411935845	Up	3.7162e-21	1.97460222987786e-20
Ifi47	99899	2916.00	6.97412774319142	Up	0	0
Hebp1	629303	1856.00	6.85798099512757	Up	1.93748e-37	1.5106289375e-36
Irf8	54123	1835.18	6.82583979831612	Up	0	0
Ifit1bl2	667373	2241.02	6.77946018060918	Up	0	0
0610010B08Rik	100039060	4539.00	6.7279204545632	Up	1.266298e-29	8.43833930400628e-29
Pydc4	100033459	2987.00	6.71169883555578	Up	0	0
Npr1	18159	1042.00	6.68650052718322	Up	1.621418e-30	1.10124010360288e-29
Il11	16153	1306.00	6.6724253419715	Up	1.334908e-08	4.30571276197719e-08
Gpr31b	436440	960.00	6.65535182861255	Up	2.37916e-27	1.49776729350649e-26
Pcyox1l	18546	669.00	6.62935662007961	Up	0.0001633958	0.000388586086739557
Oasl2	231655	2124.00	6.61228530272219	Up	0	0
Gm18853	100417831	4357.00	6.59991284218713	Up	1.385084e-49	1.33746927398976e-48
Slfn5	20558	3913.06	6.57256489558894	Up	0	0
Gp6	243816	1152.00	6.53138146051631	Up	3.44884e-24	1.99227168675519e-23
Ms4a4c	60361	1282.00	6.44294349584873	Up	3.84926e-07	1.12191281841608e-06
Rsc1a1	58185	3785.00	6.44108948924404	Up	0	0
Serpina3g	238393	2236.50	6.39476890177286	Up	5.993e-74	7.90032929292929e-73
Il23r	83430	1359.00	6.33539035469392	Up	4.7436e-25	2.81103449528467e-24
Ccr4	12773	2787.00	6.33270793364061	Up	1.784368e-62	2.05662958478261e-61
Ptgs2os	19225	4460.00	6.22570199603902	Up	0	0
Dthd1	100322896	2437.00	6.20945336562895	Up	1.60547e-11	6.01687740667109e-11
Cxcl9	17329	2905.00	6.14210705730255	Up	1.17866e-180	3.09074892735703e-179
Calcr	12311	3471.00	6.12928301694497	Up	2.5692e-15	1.13779815191856e-14
Il1bos	16176	1348.00	6.01810127353409	Up	0	0
Csf3	12985	1363.00	5.99699125012766	Up	5.4241e-71	6.94006641025641e-70
Ikzf2	60440	3101.86	5.97116031712002	Up	1.695854e-159	4.01281157099024e-158
Ifit1bl1	15957	2638.00	5.93100915952146	Up	0	0
Ccdc175	73936	2630.00	5.93073733756289	Up	2.36342e-10	8.35892927246196e-10
Isg20	100038882	756.00	5.91198082067871	Up	0	0
Hdc	15186	2415.00	5.8856139359781	Up	0	0
Il12b	16159	1275.00	5.82177398197057	Up	1.367286e-183	3.63595208087774e-182
Cxcl10	15945	1120.00	5.81804952285384	Up	0	0
Clic5	224796	5870.00	5.78135971352466	Up	9.57528e-19	4.7710484722467e-18
Amd2	100041585	3201.00	5.70043971814109	Up	1.206562e-10	4.33062623482124e-10
Ptgs2os2	320019	2994.00	5.64385618977472	Up	9.068e-10	3.10239338576326e-09
Ereg	13874	4136.00	5.6412307203679	Up	6.52896e-122	1.25022951873589e-120
Trim47	666747	2262.00	5.58496250072116	Up	3.84926e-07	1.12152747999313e-06
Cmpk2	22169	3215.00	5.53871551872756	Up	0	0
Gbp4	17472	4486.50	5.51849452885591	Up	0	0
Ifit3	15958	3949.00	5.46323605304621	Up	0	0
Thbs1	100039796	2816.00	5.44109979586504	Up	0	0
Ifit3b	15959	1998.00	5.43080481513716	Up	0	0
Cd40	21939	1685.11	5.39622540270576	Up	0	0
Tbxas1	109575	1621.00	5.39231742277876	Up	8.34172e-05	0.000204221676075036
Ermn	77767	3550.00	5.35755200461808	Up	1.77622e-09	5.99112296620278e-09
Cxcl5	20311	1656.00	5.34968621265395	Up	0	0
Gm6904	628693	898.00	5.26886685113595	Up	2.56396e-17	1.2215710575681e-16
Ifitm10	667370	2013.00	5.24492710673116	Up	3.14596e-150	7.05077375957728e-149
Csprs	114564	2719.00	5.24317398347295	Up	1.000202e-43	8.79700732607569e-43
Slfn8	76392	574.00	5.11547721741994	Up	1.147042e-09	3.90463775521669e-09
Gm14430	627914	4538.00	5.08746284125034	Up	4.62942e-10	1.61179437143443e-09
Upp1	381058	2666.00	5.08746284125034	Up	5.66652e-06	1.52358444247227e-05
CK137956	635169	2209.00	5.06608919045777	Up	1.44744e-54	1.49375103649635e-53
Ccr7	12775	1974.65	5.01429949459603	Up	0	0
Cxcl1	14825	964.00	4.99885817561045	Up	0	0
Ppfia4	76787	4882.00	4.99276843076892	Up	8.4536e-197	2.40643922147651e-195
Gbp10	626578	3528.00	4.95419631038688	Up	3.84926e-07	1.12133491002747e-06
Apol9a	223672	1482.06	4.9205655325056	Up	1.914246e-47	1.79729372639734e-46
Slamf6	27218	2688.00	4.86349800009514	Up	8.35818e-36	6.29963935495335e-35
Gm14023	100503468	1781.00	4.8005527150785	Up	0	0
Dlgap3	242667	3900.00	4.79441586635011	Up	4.18498e-44	3.71545634118263e-43
Mxd4	17858	2428.00	4.75850554804939	Up	1.479298e-189	4.06770986515397e-188
Plet1	226245	2385.00	4.75488750216347	Up	0.0001633958	0.000388858625725908
Stat5a	20849	2725.27	4.73286119583347	Up	8.29254e-157	1.92464067906977e-155
AW011738	100382	2745.00	4.71741279674481	Up	7.4925e-47	6.96917516447368e-46
Gm6034	547347	1633.00	4.70043971814109	Up	2.4762e-13	1.0019367803482e-12
Il27	209590	2488.00	4.69285322366928	Up	1.822716e-69	2.28560233968958e-68
Shmt1	330096	3754.00	4.64385618977472	Up	2.89288e-06	7.93670796895213e-06
Satb2	20230	6303.27	4.61470984411521	Up	1.293958e-15	5.80928590314898e-15
AW112010	107350	790.00	4.59411389475609	Up	0	0
Glis3	226075	7316.00	4.54843662469604	Up	1.965424e-82	2.80213307428571e-81

Slfn10-ps	20555	1881.00	4.48131040467541	Up	0	0
Susd3	71733	3255.00	4.47513293354217	Up	7.76936e-185	2.08238486192733e-183
**Timd4**	**171284**	**3153.00**	**4.4594316186373**	**Up**	**8.34172e-05**	**0.000204280631524249**
Phf11d	236451	1163.00	4.44592938461384	Up	0	0
**Milr1**	**243864**	**1075.00**	**4.40599235967584**	**Up**	**2.50504e-06**	**6.90616e-06**
Ddx60	234311	5994.00	4.39653891822944	Up	0	0
**Tpbg**	**244579**	**3212.00**	**4.39231742277876**	**Up**	**8.34172e-05**	**0.000204339621022235**
Oasl1	246727	4718.00	4.39076697121536	Up	0	0
Fam227b	75823	1858.00	4.34577483684173	Up	2.50504e-06	6.90728240533073e-06
Taf4b	21336	5036.00	4.28540221886225	Up	2.74508e-20	1.42294614359914e-19
Slc6a18	103098	3563.00	4.27301849440642	Up	7.38266e-11	2.67808872268548e-10
Pla2g16	85031	1994.00	4.26122990898705	Up	3.68666e-217	1.12496175467626e-215
Phf11b	219131	1371.00	4.24823614432312	Up	3.1655e-288	1.27265101895735e-286
Tmem141	270893	4415.00	4.24792751344359	Up	1.109944e-05	2.92320861595778e-05
Ldhb	16819	853.00	4.24364765003625	Up	0	0
Gbp9	236573	3633.00	4.2423216971835	Up	6.28592e-298	2.54527252315036e-296
**Il1b**	**16175**	**1974.00**	**4.19824818254303**	**Up**	**0**	**0**
Nts	18212	7049.00	4.16992500144231	Up	2.61478e-08	8.270387300522e-08
Tns1	21959	724.00	4.16992500144231	Up	5.83654e-05	0.000145152063383172
Olfr56	258571	3744.00	4.13876406995585	Up	2.94458e-117	5.50195421585903e-116
Fam26f	215900	1095.00	4.12553088208386	Up	8.34442e-29	5.45975432780563e-28
Id3	54167	3272.00	4.12101540096137	Up	2.96818e-14	1.25113395975155e-13
Gm13051	626316	2847.00	4.08746284125034	Up	1.665496e-12	6.51829414901961e-12
Gbp6	100702	4329.00	4.06717629399869	Up	0	0
Gbp5	229898	3021.00	4.06585080070632	Up	0	0
**Il27ra**	**246779**	**705.00**	**4.06111151430339**	**Up**	**3.41268e-27**	**2.14125476627219e-26**
Gbp2	14469	2471.00	3.99811798572451	Up	0	0
Pyurf	236312	3497.00	3.98493983381798	Up	0	0
Slfn1	108116	3526.92	3.97544874654997	Up	0	0
Oas2	23960	2011.96	3.95137058260637	Up	0	0
Gm14288	13999	428.00	3.94871077130315	Up	2.15262e-19	1.09214566148325e-18
Zfp819	240063	3549.00	3.94341647163363	Up	5.74764e-33	4.11106493423271e-32
Cav1	12389	2559.41	3.93444346308122	Up	0	0
Cdh6	12563	8185.00	3.90689059560852	Up	1.291704e-27	8.18642139110945e-27
Slc45a3	242259	4098.00	3.90689059560852	Up	0.0001633958	0.000388749564268686
Usp18	13531	2661.00	3.90689059560852	Up	0.000202654	0.000476869315395284
Gm4951	240327	2648.00	3.88234841134573	Up	3.12572e-139	6.58769758012422e-138
Mndal	381308	1788.06	3.88163434801616	Up	0	0
Oas3	246728	3873.00	3.8803385118575	Up	0	0
Gbp3	55932	2616.92	3.87954010746356	Up	0	0
Gm16223	433882	2516.00	3.877744249949	Up	5.39984e-17	2.54907305063996e-16
Fabp3	14077	669.00	3.8722947466818	Up	1.631716e-133	3.36375378566221e-132
Gbp2b	14468	2820.00	3.85798099512757	Up	2.61804e-43	2.28133881047766e-42
Ms4a6c	69774	1687.00	3.84799690655495	Up	1.143192e-67	1.39837025753425e-66
**Cxcr5**	**12145**	**2636.00**	**3.83289001416474**	**Up**	**1.298228e-07**	**3.91707918335408e-07**
Htra4	15566	3095.00	3.82288462035121	Up	1.773922e-62	2.04597965003399e-61
Rmnd5b	170765	1554.00	3.8073549220576	Up	2.39806e-07	7.10167323442136e-07
**H2-Q8**	**15019**	**1009.00**	**3.78540108838342**	**Up**	**1.673748e-100**	**2.79771512985222e-99**
Ms4a6d	73656	1606.05	3.75808284132328	Up	0	0
**H2-Q9**	**110558**	**1539.00**	**3.7405678308464**	**Up**	**6.7369e-77**	**9.09293917263325e-76**
**Cd83**	**12522**	**2140.25**	**3.71007511808157**	**Up**	**0**	**0**
Gm266	212539	1231.00	3.67914493977786	Up	1.273314e-14	5.46635762246964e-14
**Cxcl2**	**20310**	**1083.00**	**3.66794573201928**	**Up**	**0**	**0**
Gm12250	631323	2702.00	3.6561235296424	Up	1.375338e-246	4.76203765469388e-245
Slc6a4	22598	4037.00	3.64385618977473	Up	9.83956e-05	0.000239371917063378
Enpp4	224794	4558.00	3.63972830415205	Up	2.5949e-66	3.12900308457711e-65
Tmem51	235135	1602.00	3.61890983264449	Up	8.13818e-07	2.32366815684955e-06
Herpud1	67138	5279.00	3.60478747907741	Up	0	0
Selenbp1	330222	8513.00	3.58496250072116	Up	3.0172e-05	7.68154489795918e-05
**Ifih1**	**15953**	**1748.36**	**3.57469416526733**	**Up**	**0**	**0**
**Gng4**	**14706**	**3076.31**	**3.53605290024021**	**Up**	**2.17668e-08**	**6.91953398538505e-08**
4933432I03Rik	71264	1061.00	3.52356195605701	Up	1.533238e-12	6.01593799907493e-12
Rnf165	320311	8528.67	3.52356195605701	Up	2.59458e-10	9.16121629136316e-10
Syt7	118449	7116.00	3.52356195605701	Up	1.30345e-07	3.93213597083926e-07
Gm5087	328354	1921.00	3.50901364748786	Up	2.7764e-12	1.07963333486133e-11
Acod1	16365	2588.00	3.50807061738284	Up	0	0
Khk	16542	5464.00	3.50250034052918	Up	3.70192e-38	2.92260468683108e-37
Gm13363	433406	2790.00	3.50003124007901	Up	1.278782e-107	2.26469889478079e-106
**Ifi205**	**15951**	**2302.00**	**3.49558359575883**	**Up**	**0**	**0**
Uchl3	56791	1402.00	3.47061781134339	Up	0	0
**Cd200**	**17470**	**2363.00**	**3.46197253394657**	**Up**	**0**	**0**
Mis18a	100038659	971.00	3.4594316186373	Up	1.072218e-09	3.65359521751356e-09
Rgs1	57811	4210.91	3.4594316186373	Up	1.046122e-28	6.82110140353574e-28
Adgb	215772	5218.00	3.44973512002693	Up	1.199698e-211	3.56463682451839e-210
Slc29a1	269346	3904.00	3.42931163026096	Up	1.708806e-72	2.22669758801843e-71
**Ifngr1**	**66141**	**648.00**	**3.42745354964292**	**Up**	**0**	**0**
Trim34a	209387	3668.88	3.42535220202255	Up	0	0
Slc17a9	66859	3563.00	3.40808473863708	Up	2.05708e-39	1.66589113508353e-38
Fst	14313	2644.25	3.40628729025193	Up	0	0
Mx2	17857	2769.69	3.3532783874348	Up	0	0
Mir703	102465959	123.00	3.35171156391002	Up	2.39806e-07	7.09795637822749e-07
**Tnfsf12**	**22035**	**4944.00**	**3.34914956362239**	**Up**	**5.83948e-42**	**4.95858947347347e-41**
Tspan14	66109	1923.00	3.3445295238245	Up	2.20978e-15	9.80672965733717e-15
**Cd69**	**12515**	**1620.00**	**3.33211871776928**	**Up**	**9.28964e-240**	**3.1271434968254e-238**
Trim30d	244183	2771.00	3.33091687811462	Up	5.74122e-18	2.78540287446383e-17
Dgat2	67800	2251.00	3.32862274746137	Up	5.03026e-74	6.64668155451713e-73
**H2-T24**	**15042**	**2182.00**	**3.32701099518059**	**Up**	**0**	**0**
6530402F18Rik	76220	4308.00	3.32414592013718	Up	0	0

Sptssa	20739	8405.00	3.32192809488736	Up	5.83654e-05	0.000145130789447457
Fndc7	320181	4864.00	3.27301849440642	Up	2.46596e-06	6.80284184715447e-06
BC094916	545384	1294.00	3.27039779424728	Up	1.941192e-27	1.22523301607143e-26
Clec4e	56619	2519.00	3.25303401745049	Up	0	0
**H2-T22**	**15039**	**1758.00**	**3.25054160475564**	**Up**	**0**	**0**
Ak4	11639	4901.69	3.23315576281362	Up	1.072156e-104	1.86184224114637e-103
**Il10**	**22781**	**3580.00**	**3.22064475905018**	**Up**	**7.37978e-73**	**9.66090644135802e-72**
Prodh	19124	1504.00	3.21109315947196	Up	0	0
Gm12657	667250	978.00	3.19041145767754	Up	7.20302e-33	5.13904278048781e-32
AA467197	433470	672.00	3.19019906249662	Up	0	0
Gadd45b	17873	1305.00	3.17288134477372	Up	5.14114e-270	1.93402619157428e-268
Fscn1	14086	2667.00	3.1719874678992	Up	0	0
**Ly6g**	**17071**	**877.00**	**3.16397573511113**	**Up**	**5.8364e-16**	**2.65826476241611e-15**
Egln3	112407	2693.00	3.15571114222261	Up	1.383642e-30	9.41631374729242e-30
**Irgm2**	**15944**	**2227.00**	**3.15521421806687**	**Up**	**0**	**0**
Cp	12870	4050.68	3.1548654175088	Up	1.905116e-133	3.92259685145631e-132
Cacnb3	12297	2579.53	3.1079093661524	Up	0	0
Inpp4a	16323	1529.00	3.10363841966516	Up	0	0
Oas1b	246730	1889.00	3.09848701274451	Up	0	0
Hamp	84506	410.00	3.09686153925259	Up	2.04576e-07	6.09560311907271e-07
Megf9	54483	3165.92	3.08665438225418	Up	5.17556e-99	8.55000496202532e-98
2410017I17Rik	675325	1511.00	3.07166098512745	Up	2.23462e-48	2.1239531047619e-47
Bst2	69550	866.00	3.06291236092421	Up	0	0
Rtp4	20167	1994.00	3.05444778402238	Up	9.711e-09	3.15082857142857e-08
4930520O04Rik	75116	1144.80	3.04271167597141	Up	6.55282e-34	4.75107453504273e-33
Ms4a7	68774	1401.00	3.02675764231527	Up	0	0
Gm21284	100861870	3739.00	3.02236781302845	Up	1.474802e-18	7.31408673838059e-18
Osbpl10	18405	779.00	3.0168390622264	Up	2.02318e-39	1.6392202425979e-38
Dhx58	80861	2427.00	3.0157284998217	Up	0	0
Gm13546	100042926	950.84	3.0052245803435	Up	2.2089e-103	3.78547448484848e-102
Snn	20617	1248.14	2.98935275580049	Up	6.41594e-07	1.84402571641538e-06
**Cst7**	**13011**	**986.00**	**2.98891650087098**	**Up**	**2.57658e-39**	**2.08262297665555e-38**
**Slx4ip**	**20568**	**894.00**	**2.98085965100359**	**Up**	**0**	**0**
Gm6093	619715	2433.64	2.97490901903717	Up	1.170526e-20	6.14834183157895e-20
Fgf23	64654	2702.00	2.96347412397489	Up	1.360114e-22	7.5091747881549e-22
**Cd38**	**12494**	**3013.00**	**2.94890027544645**	**Up**	**0**	**0**
4930459C07Rik	74881	1207.00	2.94596016028711	Up	1.990838e-06	5.52807815188216e-06
**Cfb**	**14962**	**2766.97**	**2.93147157150146**	**Up**	**0**	**0**
Draxin	70433	5217.00	2.93073733756289	Up	1.316156e-12	5.17975010345628e-12
**Cd14**	**12475**	**1681.00**	**2.93050502427582**	**Up**	**0**	**0**
Abcb1a	18671	4977.00	2.9196578916824	Up	2.1512e-39	1.74128145038168e-38
**Ccl22**	**20299**	**1815.00**	**2.89145486223873**	**Up**	**0**	**0**
Nanos1	71950	2004.00	2.88752527074159	Up	4.2193e-16	1.93210914439946e-15
Casp4	12363	1443.00	2.88514638105136	Up	1.187508e-264	4.37033855271149e-263
Armcx4	100503043	9014.00	2.88173711838018	Up	1.665634e-60	1.87643734687915e-59
**Cxcl3**	**330122**	**1018.00**	**2.870615906513**	**Up**	**0**	**0**
Gm9895	100503337	664.00	2.86418614465428	Up	5.63872e-11	2.0608902093925e-10
Gm10432	100038713	4791.00	2.85798099512757	Up	1.847378e-05	4.7763814611399e-05
Kcnj2	16517	3695.38	2.85798099512757	Up	3.0172e-05	7.68269776377007e-05
Gem	14579	2257.00	2.8552145608362	Up	3.51228e-39	2.82413945402844e-38
Ube2c	74153	3162.00	2.84459368866584	Up	0	0
Stxbp3	74732	2265.13	2.83820871158372	Up	8.70708e-80	1.2138399283484e-78
**Ccl12**	**20293**	**537.00**	**2.81729315911372**	**Up**	**1.056106e-36**	**8.12971615063521e-36**
Acpp	56318	3172.30	2.81338679953001	Up	0	0
Filip1	70598	4207.00	2.8073549220576	Up	4.35534e-05	0.000109568058185053
**Iigp1**	**16145**	**2064.00**	**2.80617897168067**	**Up**	**1.48512e-239**	**4.98941503366336e-238**
**Ifit1**	**71586**	**5517.89**	**2.79611016488998**	**Up**	**0**	**0**
Ctla2b	13025	1029.15	2.78210612815244	Up	8.25202e-117	1.53512907149123e-115
Trim30b	20128	3771.00	2.7714427072463	Up	0	0
Efna2	13637	2153.00	2.76250068627334	Up	4.77828e-14	1.99528177405858e-13
Slfn2	237887	3549.10	2.75952639696699	Up	1.520642e-139	3.21285332154421e-138
Serpinb9b	20723	3397.00	2.75558127233355	Up	0	0
Lrrc17	68732	5163.40	2.75401128285874	Up	8.03944e-134	1.66135370328867e-132
Tmc8	233424	4316.00	2.72958606380061	Up	9.8785e-45	8.86765243386243e-44
Areg	11839	1215.00	2.72845409431069	Up	8.21758e-14	3.39881673037543e-13
**H2-Q7**	**15018**	**1401.20**	**2.72433653136142**	**Up**	**4.17618e-91**	**6.38891522813345e-90**
Ankrd33b	67434	6472.26	2.71699089440494	Up	6.36488e-118	1.1932215920442e-116
Adcy6	11512	6038.00	2.69100413813148	Up	3.40504e-71	4.3632861510574e-70
**Fcgr1**	**14129**	**2589.00**	**2.67399859417697**	**Up**	**0**	**0**
**Oas1c**	**23961**	**1847.92**	**2.6399161917659**	**Up**	**2.01502e-61**	**2.29441807516779e-60**
Gm14440	100503353	3382.00	2.62205181945638	Up	5.13658e-41	4.28663139596655e-40
**Ccl7**	**20306**	**912.00**	**2.61977941249484**	**Up**	**1.585736e-302**	**6.48279445204819e-301**
**Ccrl2**	**54199**	**1914.43**	**2.61942836443878**	**Up**	**2.31662e-285**	**9.20463112880561e-284**
**Ifi204**	**15950**	**4256.02**	**2.61833358847967**	**Up**	**0**	**0**
**Cd86**	**12524**	**2539.00**	**2.61468642088931**	**Up**	**0**	**0**
**Btnl2**	**547431**	**2563.00**	**2.60880924267552**	**Up**	**7.66072e-06**	**2.04165528620798e-05**
**Il20rb**	**16181**	**2474.67**	**2.60492729225897**	**Up**	**0**	**0**
Tpbgl	21983	3509.00	2.60432782558809	Up	1.946424e-24	1.13442217739608e-23
Siglecf	83382	1849.00	2.60029730865697	Up	0	0
**Il4i1**	**77125**	**2541.42**	**2.58804189365313**	**Up**	**4.96106e-36**	**3.76091796067918e-35**
Car13	71934	2299.00	2.58089931479404	Up	0	0
Mmp19	17386	2675.00	2.575561110531	Up	0	0
Smagp	74243	2409.52	2.57377876222848	Up	4.49538e-115	8.31718834023991e-114
E530011L22Rik	320301	3304.00	2.55458885167764	Up	7.66072e-06	2.04197604901807e-05
Dusp14	56405	1443.00	2.55117418726487	Up	4.70012e-10	1.63573817271795e-09
Hip1	69573	982.90	2.52457005828578	Up	8.25312e-99	1.36076223440233e-97
Slc31a1	239436	1906.00	2.51784830486262	Up	0.0001614662	0.000384374287806931
Sdcbp2	20971	2460.00	2.50211100169511	Up	3.1288e-254	1.12226682452431e-252
A530032D15Rik	381287	1760.00	2.4964258261195	Up	1.327124e-28	8.63012103641242e-28

Asb11	68854	1672.30	2.49317002480167	Up	2.69958e-20	1.39978833374083e-19
Psrc1	19188	906.00	2.47813067271364	Up	0	0
Acsl1	14081	3925.00	2.47072075872331	Up	0	0
Apol7c	108956	2044.00	2.45408569232978	Up	6.47594e-143	1.3872575510101e-141
**Ifit2**	**112419**	**3365.00**	**2.45251220469751**	**Up**	**2.89002e-11**	**1.06986863015492e-10**
**Tnfsf10**	**21942**	**2079.14**	**2.44336521644185**	**Up**	**1.560708e-228**	**5.05323891755725e-227**
**Stat2**	**20846**	**5176.99**	**2.42335336912705**	**Up**	**0**	**0**
**Cflar**	**12633**	**5783.79**	**2.42309346090611**	**Up**	**0**	**0**
Parp14	243771	3322.00	2.42194641455369	Up	0	0
Socs6	12702	2742.00	2.41869886720539	Up	0	0
Gbp7	229900	5604.27	2.40730360270261	Up	0	0
Il33	16184	4428.00	2.40590369030235	Up	1.163054e-193	3.26154944859504e-192
Lrrc27	74249	3657.00	2.40209844357135	Up	3.19614e-05	8.12613685598681e-05
Igflr1	16011	5854.00	2.39733549754538	Up	4.01194e-35	2.98275959859772e-34
Pde11a	23984	7717.00	2.39231742277876	Up	1.201886e-09	4.0864124e-09
Ddx58	230073	4943.00	2.39200540101123	Up	0	0
Rhpn2	434768	1206.00	2.38994651831601	Up	3.13552e-06	8.58296746047112e-06
Ell2	192657	3660.00	2.38481009959018	Up	0	0
**H2-Q6**	**110557**	**981.00**	**2.38187063534364**	**Up**	**9.54646e-54**	**9.72764206366366e-53**
4930486L24Rik	214639	1390.00	2.37637587890974	Up	3.58584e-06	9.76836246628131e-06
Krt24	268481	2735.00	2.37637587890974	Up	6.48592e-11	2.36086931388114e-10
Htr7	15559	2073.00	2.3679754624068	Up	9.6126e-12	3.64441053854749e-11
Tapbp	21354	2948.71	2.36776157084143	Up	0	0
Tmc3	192140	3216.00	2.35755200461808	Up	3.23892e-15	1.42768294933749e-14
**Il18rap**	**16068**	**1422.00**	**2.35627009102897**	**Up**	**5.0236e-68**	**6.16717782923299e-67**
Gm12505	100415914	757.00	2.35471592480133	Up	3.97406e-150	8.89497387335092e-149
Dnah2	327954	13704.00	2.35049724708413	Up	2.15058e-19	1.09143704098115e-18
Adora2a	11540	2604.00	2.34135862483626	Up	2.43816e-141	5.20324812075472e-140
Ceacam18	72431	1952.00	2.32192809488736	Up	1.431152e-06	4.01603123255045e-06
Siglece	20612	6427.00	2.32132179311719	Up	0	0
**Tarsl2**	**245126**	**957.00**	**2.28957191376387**	**Up**	**0**	**0**
Parp12	101187	3724.00	2.27603519123847	Up	6.42356e-142	1.37430162622951e-140
**Il1f6**	**329514**	**1396.00**	**2.26930116423615**	**Up**	**1.776636e-12**	**6.95005911367305e-12**
Lrrk2	192734	4601.00	2.26303440583379	Up	5.70454e-07	1.64597322517007e-06
Per2	18619	1442.00	2.26047416551523	Up	3.74684e-41	3.13920431802469e-40
Car2	12349	1807.00	2.22909477796029	Up	0	0
**Cxcl11**	**56066**	**1605.00**	**2.21987682341767**	**Up**	**4.92002e-09**	**1.62115088988153e-08**
**Stat4**	**20847**	**4402.00**	**2.20573067656764**	**Up**	**0**	**0**
Kctd12b	106931	2352.38	2.19854567938208	Up	9.22744e-26	5.56929018285308e-25
Nuf2	78373	1300.93	2.19281584139657	Up	1.316588e-38	1.04918891535932e-37
Omd	108078	3572.08	2.19226834598024	Up	0	0
**Fcgr4**	**246256**	**1250.00**	**2.18248692755096**	**Up**	**3.92378e-160**	**9.32364866666666e-159**
**Btla**	**208154**	**3232.95**	**2.18220333122075**	**Up**	**1.343948e-55**	**1.40836453168623e-54**
**Adgre1**	**13733**	**3245.00**	**2.18136294018933**	**Up**	**0**	**0**
Serpinb10	20715	2030.00	2.17408862638385	Up	1.316132e-149	2.91888830222222e-148
Gm12504	623796	2231.00	2.16992500144231	Up	3.26478e-09	1.08736273027091e-08
**Il6**	**14204**	**2059.00**	**2.16452336993905**	**Up**	**0**	**0**
**Sp110**	**20684**	**2517.07**	**2.1530009004648**	**Up**	**0**	**0**
Tcaf2	74413	5370.86	2.14974711950468	Up	2.51814e-12	9.80104685478321e-12
Epsti1	108670	1853.99	2.14342991122291	Up	3.24386e-300	1.31663465933014e-298
**Ramp1**	**241308**	**6141.78**	**2.14336417517118**	**Up**	**6.266e-43**	**5.42946659856997e-42**
**Jak2**	**16449**	**5493.00**	**2.13731232435387**	**Up**	**7.89696e-193**	**2.19639054688524e-191**
Parp9	547253	7251.00	2.13158008986682	Up	0	0
**H2-M2**	**14990**	**1528.00**	**2.12456795349079**	**Up**	**0**	**0**
Pnp2	18950	2941.00	2.12200944127276	Up	0	0
**Ly6c1**	**110454**	**965.96**	**2.11992817585448**	**Up**	**8.80448e-90**	**1.33015857239537e-88**
Acsbg1	94180	2775.00	2.11703942119022	Up	5.99328e-21	3.16865031100031e-20
**H2-Eb2**	**381091**	**3380.00**	**2.1057946640226**	**Up**	**3.70236e-09**	**1.22852023782515e-08**
Cpd	12874	9152.00	2.09531897642941	Up	0	0
**Arhgap28**	**268970**	**5317.00**	**2.09515723304034**	**Up**	**5.69784e-13**	**2.27083752501762e-12**
**Ly6c2**	**17067**	**744.91**	**2.08065766334522**	**Up**	**1.435926e-08**	**4.61749820242608e-08**
Gm10033	378466	1759.87	2.07800251200127	Up	1.519112e-14	6.49855123348462e-14
Slc16a7	65221	2345.00	2.07661764433194	Up	0	0
Chst1	76969	2682.00	2.07528812730424	Up	1.850142e-43	1.62135894483471e-42
**Racgap1**	**170758**	**1078.00**	**2.05428386934392**	**Up**	**3.95986e-17**	**1.87714402794077e-16**
**H2-T10**	**15024**	**2895.36**	**2.05277703103786**	**Up**	**1.863468e-56**	**1.97844794042553e-55**
**Isg15**	**54396**	**3537.00**	**2.0487930990846**	**Up**	**4.0571e-166**	**9.932576998557e-165**
Sepw1	20361	3290.00	2.04592191261877	Up	3.9176e-86	5.79476910200523e-85
Parp11	671535	3331.08	2.0435690149374	Up	7.65442e-223	2.40490536518518e-221
Tmem171	66817	1943.00	2.03394733192334	Up	7.41494e-05	0.000182612675337495
**Adgrg6**	**215798**	**6505.00**	**2.03180002817129**	**Up**	**6.39152e-150**	**1.42494780972405e-148**
**Fmnl2**	**71409**	**5851.00**	**2.0215585653591**	**Up**	**0**	**0**
Nlrp10	434341	6945.00	2.01731458530217	Up	1.989592e-213	5.97441024283186e-212
**Tnfrsf8**	**21938**	**4913.00**	**2.01564419892893**	**Up**	**0**	**0**
**Sh3gl2**	**24056**	**2487.00**	**2.012025868807**	**Up**	**7.74134e-192**	**2.14958386972177e-190**
Arg2	11847	1417.00	1.99556775368474	Up	7.92216e-40	6.4650007965368e-39
**Ly75**	**57248**	**878.00**	**1.99443284676923**	**Up**	**1.278942e-209**	**3.76057711819757e-208**
Pappa	329217	3917.00	1.99117614961299	Up	1.005222e-14	4.3296766824067e-14
Col27a1	373864	7635.00	1.98606080904213	Up	2.6491e-18	1.30048120949074e-17
**S1pr4**	**13610**	**4484.00**	**1.98569216331612**	**Up**	**2.56046e-58**	**2.79002982402055e-57**
4933412E12Rik	71086	1559.61	1.98063467513307	Up	6.20604e-11	2.25996296716033e-10
Slc25a39	67712	4162.00	1.97515864517204	Up	1.931104e-225	6.14692504015009e-224
**H2-Q5**	**15016**	**1181.00**	**1.9608049371989**	**Up**	**5.1102e-52**	**5.10899547436653e-51**
Dtx3l	209200	5181.00	1.94832713351753	Up	0	0
Serpinb9	20708	1859.00	1.94535443363507	Up	7.60156e-73	9.94356722898997e-72
Socs3	12703	1226.72	1.93614221202207	Up	1.540576e-264	5.65744857489177e-263
Pp2d1	18996	3801.00	1.93288580414146	Up	0.0001191406	0.000287694195787077
Spta1	74646	3089.00	1.93246684231453	Up	1.305924e-40	1.07816577051095e-39
Ccnd2	12444	5772.00	1.9305907621611	Up	0	0

Gm20300	100504586	3459.00	1.91886323727459	Up	5.2379e-27	3.267140125e-26
Zfp811	210105	4993.00	1.91276716194519	Up	1.867486e-38	1.4812420512389e-37
Ppa2	110332	2476.00	1.91253715874966	Up	3.90494e-14	1.63623640503828e-13
Msantd3	109225	1336.00	1.91104907709025	Up	2.76188e-49	2.65484736997167e-48
**Cd300e**	**217306**	**1963.00**	**1.90332398058833**	**Up**	**3.82834e-09**	**1.26933000664452e-08**
Gm8221	666661	8431.00	1.89812038598079	Up	2.4986e-08	7.91767792304819e-08
Perp	74183	3849.00	1.89649542424614	Up	1.815004e-38	1.44163660411985e-37
Trim16	319236	3736.00	1.88258468909239	Up	2.25774e-114	4.15453544902386e-113
Cd274	60533	3653.00	1.87750757909205	Up	0	0
**Ccl2**	**20296**	**806.00**	**1.8713229512601**	**Up**	**0**	**0**
Nr4a3	18227	3098.85	1.87101355927992	Up	3.903e-10	1.36504427952999e-09
Tmeff1	73130	3751.00	1.86810399458684	Up	0	0
Zranb3	98999	6191.25	1.86097913445711	Up	0	0
Serf1	20364	722.00	1.84686734663063	Up	5.28728e-216	1.60472258461538e-214
Pak1	18600	4758.00	1.84549005094438	Up	6.46726e-54	6.61383563351416e-53
Sapcd2	209086	5344.00	1.84074296145954	Up	0	0
Flt4	14257	5853.00	1.83650126771712	Up	5.86706e-10	2.02936880652395e-09
Gja5	14613	3180.00	1.83550596237175	Up	2.60094e-26	1.5856107811714e-25
Trpv4	107328	1120.74	1.83212282720655	Up	5.93614e-06	1.5933009213732e-05
Gm29811	101243624	1668.01	1.82851953205565	Up	9.2937e-08	2.8354057579572e-07
Parpbp	80285	3564.00	1.82043839891152	Up	0	0
Serpinb6b	18788	1993.58	1.81792651068381	Up	0	0
1600014C10Rik	72244	3205.96	1.8124437987304	Up	6.25042e-237	2.09160997475345e-235
Sp140	109032	1921.31	1.79907500431412	Up	0	0
**Tnfrsf9**	**21941**	**1575.00**	**1.79399653920259**	**Up**	**1.44333e-07**	**4.34022275434243e-07**
Abca1	11303	10260.00	1.78884869236286	Up	0	0
Sorbs3	20656	3824.00	1.77852962068008	Up	0	0
Msmo1	76626	6505.00	1.77760757866355	Up	5.1886e-10	1.80167391731478e-09
Tfeb	81879	9285.00	1.77760757866355	Up	3.84698e-05	9.72115917187965e-05
**Trim25**	**20821**	**2785.06**	**1.77725043172748**	**Up**	**3.8572e-89**	**5.81700046222222e-88**
Glrp1	14659	1957.00	1.77610398807316	Up	7.43756e-16	3.36764459460902e-15
Arg1	11846	1489.00	1.7723667798454	Up	8.65428e-172	2.1947461058296e-170
Tyro3	22173	5894.00	1.7714539705515	Up	2.84428e-69	3.5534649837997e-68
Bcl2a1d	12047	796.00	1.76342482670221	Up	0	0
Psme1	16912	806.00	1.7590036106357	Up	3.12306e-132	6.36084465306122e-131
**Il23a**	**213208**	**2479.00**	**1.75716916488939**	**Up**	**2.59108e-105**	**4.5180126700925e-104**
Hspa1b	193740	2798.00	1.7376459502462	Up	2.76178e-36	2.10401254961832e-35
Batf2	74481	1433.00	1.73696559416621	Up	3.78974e-32	2.66902153756746e-31
Cyp26b1	232174	4700.00	1.73315398021619	Up	1.17788e-104	2.04125761797753e-103
Ppp1r9a	54646	3489.00	1.72935241005633	Up	3.84698e-05	9.72260728139431e-05
Thbs4	21825	5863.69	1.72391208329133	Up	0	0
Casp1	12362	1533.00	1.7218998969707	Up	1.161556e-279	4.51994474678899e-278
Gpr18	110168	1334.00	1.71961202741594	Up	2.94802e-08	9.2777049378594e-08
1700100I10Rik	74982	838.00	1.71781449382129	Up	2.66718e-14	1.12621642309607e-13
Trim30a	217069	5596.00	1.71575966001928	Up	0	0
Jam2	16452	5026.44	1.71480056048951	Up	0	0
Gm20362	100504701	2082.84	1.70110409533031	Up	1.47562e-39	1.19729167479675e-38
Aoah	27052	2045.20	1.68876374909915	Up	4.51764e-223	1.42200890983302e-221
Fyb	23880	4997.00	1.68737167279923	Up	0	0
Penk	67245	3501.00	1.68589140957194	Up	3.40516e-88	5.09902423300971e-87
Plscr4	229791	5690.00	1.6828098241193	Up	4.2635e-07	1.23902947927372e-06
Ndrg3	17988	2909.00	1.68247900663542	Up	9.89698e-135	2.06027193472393e-133
**Cxcl16**	**66102**	**2448.00**	**1.67801988892252**	**Up**	**0**	**0**
Zcchc24	244871	6133.00	1.67548196648549	Up	5.47622e-149	1.20818658673602e-147
Srm	19073	938.00	1.66776602467238	Up	0	0
Mri1	381269	2493.00	1.66464393184696	Up	3.822e-60	4.28013544554455e-59
Tor3a	240832	3164.55	1.66248773141472	Up	0	0
Fnbp1l	214459	5206.93	1.66051353372833	Up	5.37636e-94	8.43025173382625e-93
Rapgef5	76089	6513.95	1.65839794732417	Up	9.84838e-131	1.98205949086595e-129
Rem1	19696	2584.00	1.65050427676094	Up	9.16384e-62	1.04837295643965e-60
Glrx	93692	1335.00	1.64970152987536	Up	0	0
Grap	71520	1599.00	1.64711897678373	Up	1.792318e-14	7.63264738654619e-14
Dnmt3l	54427	1283.12	1.63785219342407	Up	4.51788e-25	2.68008224055944e-24
Car4	12351	1316.00	1.63453333230495	Up	0	0
Rnd1	68195	1031.00	1.62670500128027	Up	3.8265e-167	9.43610450581395e-166
Bcr	110279	6537.00	1.62358379474846	Up	3.62432e-29	2.39354663760218e-28
Slamf7	30925	2443.00	1.61186338391462	Up	4.5581e-80	6.37532766694147e-79
Pdzd9	68070	13413.00	1.58496250072116	Up	2.68724e-07	7.91935276011812e-07
Gm15645	626055	1547.00	1.5829739476277	Up	1.68153e-18	8.3198710936133e-18
Spata31d1b	219140	5502.02	1.5782561781704	Up	1.698842e-145	3.68574851304348e-144
Arid5a	214855	5679.53	1.57650092197296	Up	2.54962e-31	1.76270794295029e-30
**Rasgrp2**	**19419**	**5155.00**	**1.57623209561662**	**Up**	**1.14525e-259**	**4.15177596153846e-258**
Slpi	380773	427.00	1.5716656781077	Up	2.83552e-69	3.54513134266765e-68
**H2-T23**	**15040**	**1317.00**	**1.57057556273873**	**Up**	**0**	**0**
Mcoln3	68279	2514.58	1.56571417563459	Up	4.10774e-150	9.18207072990777e-149
**Irf9**	**15900**	**2855.53**	**1.56006398174379**	**Up**	**0**	**0**
Psme2	19186	977.00	1.55057309503866	Up	0	0
Perm1	18627	5845.00	1.54477341060546	Up	1.81469e-47	1.70476359579181e-46
Daxx	13163	2604.32	1.54448215367184	Up	3.06696e-172	7.78952744910179e-171
Gm14295	100039123	1324.00	1.54272456800849	Up	1.491824e-56	1.58784730138018e-55
**Tlr11**	**21897**	**2872.88**	**1.54260510847186**	**Up**	**2.08076e-69**	**2.60532650627306e-68**
Tgtp1	21817	3549.00	1.53960614001126	Up	0	0
Gm4759	209380	3091.00	1.53051471669878	Up	2.41984e-06	6.67995532704198e-06
Fpr1	14293	1332.00	1.53043875066907	Up	0	0
**Itgae**	**16402**	**4440.19**	**1.52564750305286**	**Up**	**0**	**0**
Mtmr7	17769	5895.96	1.52252192594525	Up	9.90236e-191	2.73621237394137e-189
Znfx1	574428	2801.00	1.51708083535985	Up	1.060914e-90	1.61720277843666e-89
Clic4	29876	4065.00	1.51684479559468	Up	0	0

H2-Q4	15015	1793.00	1.5141373836666	Up	0	0
Tnfaip3	21926	1653.00	1.51355826245312	Up	0	0
Il12a	16156	1870.55	1.51019473231918	Up	2.65988e-06	7.32233069608957e-06

Supplemental Table S3: Up and down regulated genes in non-stimulated BMDMs treated with ABX464

Symbol	Gene ID	Gene Length	log2 Ratio (NS-ABX464/NS)	Up and Down-Regulation (NS-ABX464/NS)	P-value	FDR
Cyp1a1	13076	2637.00	6.59991284218713	Up	5.7617e-16	9.4190291015625e-15
Mpzl2	14012	3216.00	2.22571277962806	Up	1.019862e-12	1.29631661958998e-11
Cyp1b1	13078	5128.00	1.66190264519218	Up	4.25344e-155	1.29459246545455e-152
Serpinb2	18788	1991.44	1.63788917287641	Up	0	0
Mefv	54483	3139.86	1.55254102302878	Up	1.83494e-15	2.87073790654206e-14
Cck	12424	708.78	1.54919962224722	Up	1.411874e-160	4.45939070943396e-158
Gm7367	664849	1401.00	1.45124531877809	Up	4.54926e-14	6.40492955424727e-13
Gm8221	666661	8431.00	1.44745897697122	Up	7.7881e-05	0.000389172519402985
Tiparp	99929	4154.00	1.21810007149909	Up	2.45064e-112	4.60940602247191e-110
Hic1	15248	3506.43	1.20163386116965	Up	2.35592e-09	2.17529513513514e-08
Asb2	65256	2707.00	1.17293375131465	Up	5.02582e-30	1.752754725e-28
Sptbn2	20743	8254.00	1.14295795384204	Up	2.5075e-05	0.000136240019474197
Ctla2b	13025	1005.25	1.09405013446718	Up	4.36764e-11	4.81648837944664e-10
Apol7c	108956	2044.00	1.01841763362973	Up	1.666358e-16	2.79227556756757e-15
Rn7s2	103949	299.00	-3.16394402439158	Down	4.52462e-18	8.39713290465632e-17
Rn7s1	103948	299.00	-3.16394402439158	Down	4.52462e-18	8.38783375415282e-17
Atp6-ps2	100039636	1133.00	-3.02974734339405	Down	2.51252e-34	1.0233475620438e-32
Pla2g4b	211429	3859.00	-2.93073733756289	Down	1.576718e-09	1.48951802031603e-08

Supplemental Table S4: Up and down regulated genes in LPS-stimulated BMDMs treated with ABX464

Symbol	Gene ID	Gene Length (kbp)	log2 Ratio (LPS-ABX/LPS)	Up-Down-Regulation (LPS-ABX/LPS)	P-value	FDR
Il22	50929	1088.00	7.57742882803575	Up	2.87184e-13	5.02691163485477e-12
Rtn4rl2	269295	1263.00	3.15919859484925	Up	8.91886e-38	4.70302636375e-36
Mpzl2	14012	3216.00	3.10080064078749	Up	2.05616e-20	5.6051120904685e-19
Cck	12424	708.51	2.54740573462801	Up	4.73742e-161	1.6654005225e-158
Hic1	15248	3444.08	2.09686153925259	Up	4.62354e-13	7.94476720570265e-12
Asb2	65256	2707.00	2.05658352836637	Up	2.79168e-20	7.5370893312e-19
Il12rb1	16161	2873.00	2.01526675665331	Up	2.7193e-13	4.77476255983351e-12
Cyp1b1	13078	5128.00	1.98069769922713	Up	3.42816e-300	2.41028216e-297
Rgs9	19739	6163.67	1.93586966258028	Up	1.873932e-10	2.59611893004926e-09
Tnfrsf13c	72049	1906.00	1.74474294475793	Up	1.189976e-05	8.79529348401226e-05
Ifitm1	68713	698.46	1.67324632049559	Up	1.44787e-118	3.09257701012658e-116
Ido2	209176	3014.00	1.65711228647699	Up	8.54564e-12	1.343887505685e-10
Gm8221	666661	8431.00	1.53726575868986	Up	1.027648e-16	2.26970318743455e-15
Naip7	53880	4841.00	1.53343220008107	Up	3.4085e-05	0.000231169730707396
Nqo1	18104	1552.00	1.4192599032045	Up	6.23092e-158	2.14572538938775e-155
Dnah2	327954	13704.00	1.25831199559139	Up	4.77998e-20	1.27420825466035e-18
Amica1	270152	2472.00	1.17512611315022	Up	8.95934e-59	8.0844868e-57
0610010B08Rik	100039060	4539.00	-6.7279204545632	Down	4.65744e-30	1.88013498947368e-28
Gm14430	627914	4538.00	-5.08746284125034	Down	3.32796e-10	4.49967925e-09
Ckap2	80986	2601.00	-1.20645087746743	Down	2.3192e-05	0.000162046297308489
Rnase2a	93726	722.00	-1.11263743005075	Down	1.185492e-10	1.66978230450751e-09

Supplemental Tale S5 : Up and down regulated genes in common between LPS-stimulated and non-stmulated BMDMs treated with ABX464

Symbol	Gene ID	Gene Length	log2 Ratio (NS-ABX464/NS)	Up and Down-Regulation (NS-ABX464/NS)	P-value	FDR
Mpzl2	14012	3216.00	3.10080064078749	Up	2.05616e-20	5.6051120904685e-19
Cyp1b1	13078	5128.00	1.98069769922713	Up	3.42816e-300	2.41028216e-297
Cck	12424	708.51	2.54740573462801	Up	4.73742e-161	1.6654005225e-158
Gm8221	666661	8431.00	1.53726575868986	Up	1.027648e-16	2.26970318743455e-15
Hic1	15248	3444.08	2.09686153925259	Up	4.62354e-13	7.94476720570265e-12
Asb2	65256	2707.00	2.05658352836637	Up	2.79168e-20	7.5370893312e-19

Gene designation	Forward (5′ ->3′)	Reverse (5′ ->3′)
Gus	GATTCAGATATCCGAGGGAAAGG	GCCAACGGAGCAGGTTGA
hprt	GCAGTACAGCCCCAAAATGG	GGTCCTTTTCACCAGCAAGCT
Tbp	ACTTCGTGCAAGAAATGCTGAAT	CAGTTGTCCGTGGCTCTCTTATT
Il 22	AGCTTGAGGTGTCCAACTTC	CCGGACATCTGTGTTGTTATCT
Il 10	ACAGCCGGGAAGACAATAAC	CAGCTGGTCCTTTGTTTGAAAG

Supplemental Table S6. List of primers used in qRT-PCR studies

Supp Figure S1

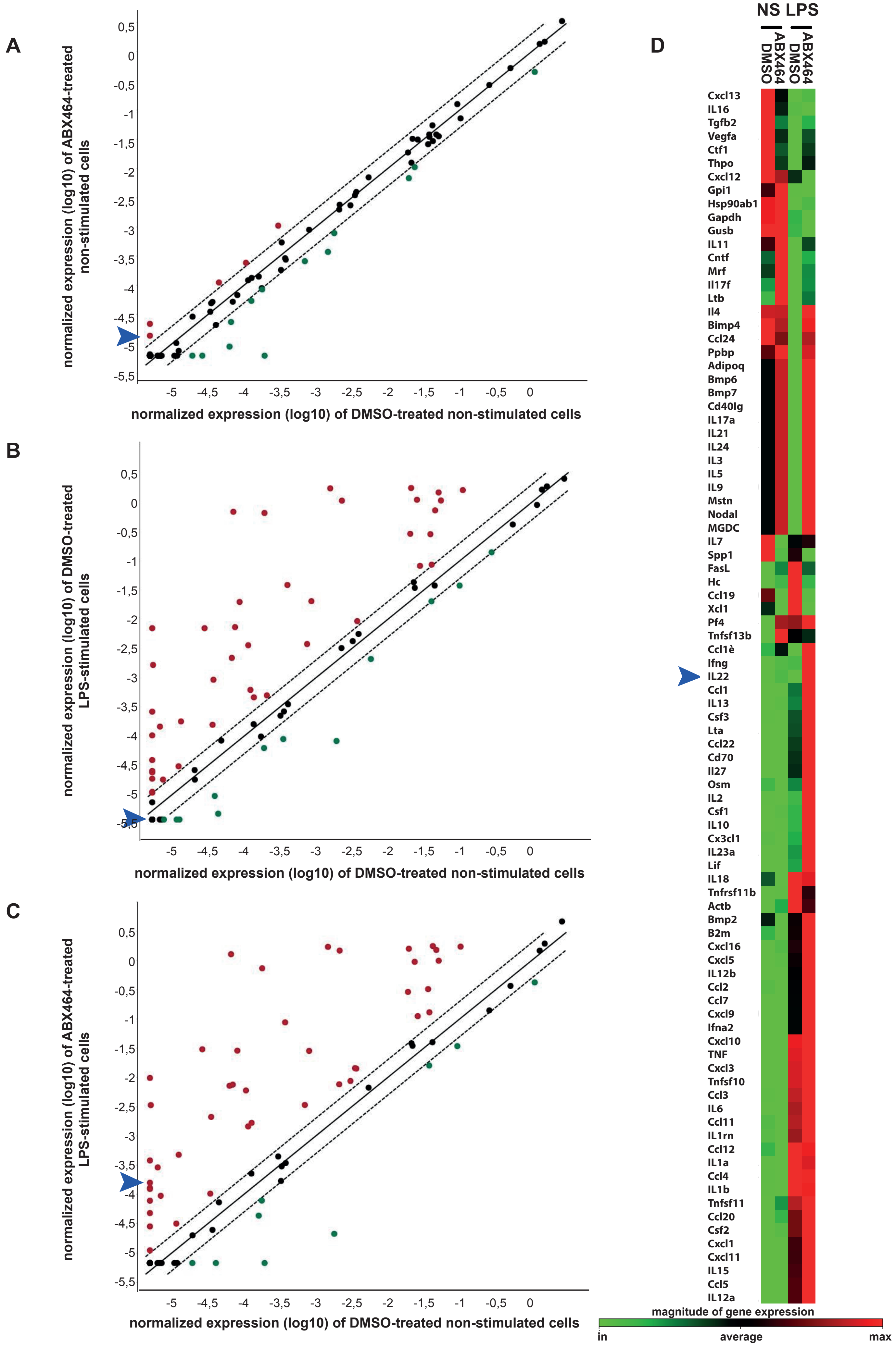

ABX464 treatment induced up regulation of IL22 in LPS-stimulated BMDMs .

Identification of cytokines and chemokines regulated by ABX464 in BMDMs. Bone marrow isolated cells were cultured for 6 days in the presence of GM-CSF (50ng/ml) to differentiate into macrophages. RNA was isolated from cells were kept in culture for additional 3 days in the presence of ABX464 (5μM) or vehicle (DMSO) alone and for additional 6 hours stimulated with LPS (4μg/ml). Scatterblots correlate gene expression of ABX464-treated cells (A), DMSO (DMSO)-treated cells and LPS-exposed cells (B) and ABX464-treated and LPS-treated cells (C) versus DMSO only treated cells. Samples represent the pool of RNA samples isolated from BMDMs derived from 3 different mice . (D) The clustergram shows differential gene expression between DMSO- and ABX464-treated cells as monitored by RT2 profiler PCR array (Qiagen). Arrow heads indicate expression level of IL-22.
